# Heterometallic Dual-Liganded AE-Ln-CPs Luminescent Probes for Efficient Sensing of Fe(III) Ions

**DOI:** 10.3389/fchem.2022.865447

**Published:** 2022-04-07

**Authors:** Jieqiong Hou, Yanmei Chen, Shuixiang Zou, Wenwen Dong, Zhenghua Ju, Junqi Lin, Zhijun Ruan, Shanshan Liu, Zhengfang Tian

**Affiliations:** ^1^ Hubei Key Laboratory for Processing and Application of Catalytic Materials, College of Chemistry and Chemical Engineering, Huanggang Normal University, Huanggang, China; ^2^ College of Materials and Chemical Engineering, Key Laboratory of Inorganic Nonmetallic Crystalline and Energy Conversion Materials, Hubei Provincial Collaborative Innovation Center for New Energy Microgrid, China Three Gorges University, Yichang, China; ^3^ Analysis and Testing Center, Lanzhou University, Lanzhou, China

**Keywords:** lanthanide complex, coordination polymer, fluorescent probes, fluorescence sensing, sensing Fe(III) ion

## Abstract

Iron ion is widely present in the environment and in biological systems, and are indispensable trace elements in living organisms, so development of an efficient and simple sensor for sensing Fe(III) ions has attracted much attention. Here, six heterometallic AE-Ln coordination polymers (CPs) [Ln_2_ (pda)_4_(Hnda)_2_Ca_2_(H_2_O)_2_]·MeOH (Ln = Eu (**1**), Tb (**2**); H_2_pda = 2,6-pyridinedicarboxylic acid, H_2_nda = 2,3-naphthalenedicarboxylic acid), [Ln (pda)_2_ (nda)AE_2_(HCOO)(H_2_O)] (AE = Sr, Ln = Eu (**3**), Tb (**4**); AE = Ba, Ln = Eu (**5**), Tb (**6**)) with two-dimensional (2D) layer structures were synthesized by hydrothermal method. All of them were characterized by elemental analysis, XRD, IR, TG, as well as single crystal X-ray diffraction. They all show infinite 2D network structure, where complexes **1** and **2** are triclinic with space group of $P_{\bar{1}}$ , while **3**-**6** belong to the monoclinic system, space group *P*2_1/*n*_. The solid-state fluorescence lifetimes of complexes **1**, **3** and **5** are τ_obs1_ = 1930.94, 2049.48 and 2,413.04 µs, respectively, and the quantum yields *Ф*_total_ are 63.01, 60.61, 87.39%, respectively, which are higher than those of complexes **2**, **4** and **6**. Complexes **1**-**6** all exhibited efficient fluorescence quenching response to Fe^3+^ ions in water, and were not interfered by the following metal ions: Cu^2+^, Cd^2+^, Mg^2+^, Ni^2+^, Co^2+^, Ca^2+^, Ba^2+^, Sr^2+^, Li^+^, Na^+^, K^+^, Al^3+^, Fe^2+^, Pb^2+^, Cr^3+^, Mn^2+^ and Zn^2+^. The quenching coefficient *K*_SV_ for complexes **1-6** is 1.41 × 10^5^ M^−1^, 7.10 × 10^4^ M^−1^, 1.70 × 10^5^ M^−1^, 1.57 × 10^5^ M^−1^, 9.37 × 10^4^ M^−1^, 1.27 × 10^5^ M^−1^, respectively. The fluorescence quenching mechanism of these complexes towards Fe^3+^ ions was also investigated. It is possible that the weak interaction formed between the complexes and the Fe^3+^ ions reduce the energy transfer from the ligand to the Ln^3+^ ion, producing the emission burst effect. This suggests that complexes **1**-**6** can be candidate for efficient luminescent sensor of Fe^3+^.

## Introduction

In recent decades, the problem of water pollution has become increasingly serious, especially the contamination of heavy metal ions in water has caused widespread concern among chemists, as they are closely related to our health (Jia et al., 2020). Iron ions are widely present in biological and water systems and are indispensable trace elements in living organisms (Gogoi et al., 2019). It is the main component of hemoglobin and plays an important role in oxygen uptake (D'Autreaux et al., 2005), release, and transport; in addition, it is involved in oxidation-reduction reactions and the transport of protons and electrons (D'Autreaux et al., 2005) in living organisms and plays an important function in the respiratory chain. Each coin has two sides, and excess or deficiency of Fe(III) ions may lead to serious diseases (Brugnara, 2003) such as: iron deficiency anemia (Bricks et al., 2005), aplastic anemia, decreased immunity (Barba-Bon et al., 2012), impaired behavioral and intellectual development, and liver fibrosis. Therefore, selective detection of Fe(III) ions is very important for human health.

The traditional methods for the qualitative or quantitative analysis of Fe(III) ions include atomic absorption spectrophotometry (AAS), inductively coupled plasma atomic emission spectrometry (ICP-AES), colorimetric methods, fluorescence spectrophotometry and electrochemical methods (B. Fan et al., 2016). However, these methods suffer from cumbersome sample pretreatment procedures, time-consuming detection processes, high costs (Dang et al., 2013), and lack of portability. Compared with the traditional platform detection, the chemical sensor based on fluorescence detection has simple operation, short time consumption, low cost, and can meet the requirements of real-time detection. Therefore, it is essential to develop a new cheap, highly selective, sensitive, and accurate chemical sensor for the detection of Fe(III) ions in biological and aqueous systems.

As an attractive multifunctional luminescent material, coordination polymers (CPS) have attracted great interest due to their structural diversity and potential applications in gas storage/separation (Chen et al., 2006; Getman et al., 2012), sensing (Parmar et al., 2017; Wu et al., 2017; Qin et al., 2018; Hu et al., 2020; Tian et al., 2020; Yao et al., 2021), luminescence (Cui et al., 2012), catalysis (Zhou et al., 2019), magnetism (Yue and Gao, 2019), ion exchange (Chen et al., 2011), photoreduction (Wang X.-K et al., 2019), and so on. Among these coordination polymers, lanthanide coordination polymers (Ln-CPs), especially Tb(III)-CPs and Eu(III)-Cps, are used as highly sensitive and selective fluorescent probes because of their outstanding luminescence characteristics, such as large Stokes shift, narrow emission spectrum, excellent quantum yield, high color purity and extended emission life due to antenna effect. They are used for the sensing of different types of analytes, including small molecules (Qin et al., 2018), metal cations (Zhao et al., 2016), inorganic and organic anions (Liu et al., 2016), solvents (Zhang et al., 2018), gases (Wang W et al., 2019; Xie et al., 2020), and explosives (Jiang et al., 2020). In recent years, various luminescent Ln-CPs fluorescent sensors have been significantly developed in sensing metal ions (Liu et al., 2018; Lv et al., 2020; Ma et al., 2020; Esrafili et al., 2021). The fluorescence quenching of Ln-CPs can recognize iron (III) ions (He et al., 2022). However, there are two problems in the process of detecting iron (III) ions: 1) poor selectivity. Other metal ions such as Cu (II), Al (III) and Fe (II) can also quench the fluorescence of Ln-CPs, thus interfering with the detection of Fe (III). 2) Poor stability. Most Ln-CPs have low thermal and chemical stability and are easy to decompose in water, which limits the application of Ln-CPs in practical detection. Therefore, it is still a challenge for chemists to design and synthesize new Ln-CPs fluorescent probes with high stability, which can recognize Fe (III) ions in an efficient and specific way and establish a good correlation with iron (III) ion concentration.

The factors affecting the structure and luminescence properties of Ln-CPs mainly include internal factors (the properties of organic ligands (Chen et al., 2017) and the coordination mode of metal ions (Gai et al., 2017) and external factors (solvent type/polarity (Masoomi et al., 2017), pH of reaction (Rowsell and Yaghi, 2004), etc.). Among these factors, the most important is to select ligands with π-conjugation effect (Reineke et al., 2000; He et al., 2018), and the resulting antenna effect makes it easy to transfer energy to Ln (III) center. Pyridine-2,6-dicarboxylic acid ligand has been proved to be an ideal N,O-chelating ligand. The antenna effect produced by its π-conjugation system can sensitize the luminescence of Ln (III) (Tao et al., 2015; Tong et al., 2020). First, we chose pyridine-2,6-dicarboxylic acid and 2,3-nedicarboxylic acid as organic ligands. They have π-conjugation effect and multiple carboxyl groups, and when they are coordinated with Ln (III) ions, they can exclude the coordination of water molecules with the central Ln (III) ions and effectively sensitize the luminescence of Ln (III) ions, showing a better luminescence effect. They can be used for selective recognition of Fe(III) ions by forming weak interactions with Fe(III) ions through uncoordinated carboxylate oxygen atoms. Secondly, we chose alkaline earth metal ions (Ca(II)/Sr(II)/Ba(II)) as the bridging metal ions to synthesize bimetallic AE-Ln-CPs. The alkaline earth metal ions have the effect of enhancing the fluorescence performance of Ln (III) complexes, which is beneficial to improve the luminescence lifetime and quantum yield of the complexes. And the ionic potential of alkaline earth metal ions is small, which can improve the stability of the complexes.

In this paper, we have successfully synthesized and characterized six new AE-Ln-CPs, namely [Ln_2_ (pda)_4_(Hnda)_2_Ca_2_(H_2_O)_2_]·MeOH (Ln = Eu (**1**), Tb (**2**)), [Ln(pda)_2_(nda)AE_2_(HCOO)(H_2_O)] [AE = Sr, Ln = Eu (**3**), Tb (**4**); AE = Ba, Ln = Eu (**5**), Tb (**6**)]. As expected, the luminescence results show that complexes **1**, **3** and **5** have high fluorescence lifetime and quantum yield. Complexes **1**-**6** all can be used as fluorescence probes for Fe (III) ions in aqueous solution, and have strong quenching effect, high selectivity, and sensitivity (1.41 × 10^5^ M^−1^, 7.10 × 10^4^ M^−1^, 1.70 × 10^5^ M^−1^, 1.57 × 10^5^ M^−1^, 9.37 × 10^4^ M^−1^, 1.27 × 10^5^ M^−1^, respectively, FeCl_3_). The possible fluorescence sensing mechanism is also discussed in detail.

## Experimental Section

### Materials and Measurements

Commercially obtained reagents and solvents were used. The C, H and N microanalyses were carried out with a Carlo-Erba EA1110 CHNO-S elemental analyzer. FT-IR spectra was recorded from KBr pellets in the range of 4,000–400 cm^−1^ on a Nicolet MagNa-IR 6700 spectrometer. Powder X-ray diffraction (PXRD) patterns was recorded on a SHIMADZU XRD-6100 diffractometer at 40 kV and 30 mA with a Cu-target tube and a graphite-monochromator. Crystal determination was performed with a Bruker SMART APEX-ΙΙI CCD diffractometer. Thermal gravimetric analysis (TGA) was conducted on a SDT Q600 instrument in flowing N_2_ with a heating rate of 10 °C/min. The solid-state luminescence emission/excitation spectra were recorded on a RF-5301 fluorescence spectrophotometer. The solid-state luminescence quantum yields, and lifetimes were measured on an Edinburgh FLS920 combined fluorescence lifetime and steady state spectrometer.

### X-Ray Crystal Structure Determination

Complexes **1**-**6** were collected by means of *ω*-2*θ* scanning on a Bruker SMART APEX-III CCD diffractometer with graphite-monochromated Mo*Kα* radiation (*λ* = 0.71073 Å). All structures were solved by direct methods, and SHELXS-2014 (Sheldrick, 2014) and SHELXL-2016 (Sheldrick, 2015) are used to refine *F* ^*2*^ with full-matrix least-squares.

### Synthesis of Complexes 1-6

A mixture of Ln (NO_3_)_3_·6H_2_O (Ln = Eu (**1**), Tb (**2**), 0.05 mmol, 22.4 mg), CaCl_2_ (0.10 mmol, 11.1 mg), H_2_nda (0.20 mmol, 43.2 mg), H_2_pda (0.20 mmol, 33.2 mg) and 2 ml H_2_O plus 5 drops of DMF were sealed in a Pyrex-tube (10 ml), which was heated at 120°C for 2 days. After cooling to room temperature, colorless and transparent strip crystals were obtained, washed with water, and dried at room temperature. The synthesis method of **3**-**6** is the same as above, except that CaCl_2_ is replaced by SrCl_2_.6H_2_O (0.1 mmol) or BaCl_2_.2H_2_O (0.1 mmol), and H_2_O/DMF (v:v = 3:1, 2 ml) was used as the solvent. All these crystals are insoluble in water. Their yields based on Ln (NO_3_)_3_·6H_2_O were 44.1% (**1**), 49.8% (**2**), 38.4% (**3**), 38.7% (**4**), 71.2% (**5**), 63.0% (**6**), respectively. IR data and element analysis see SI part 1.

## Results and Discussion

### Structural Description of Complexes 1-2

**FIGURE 1:**
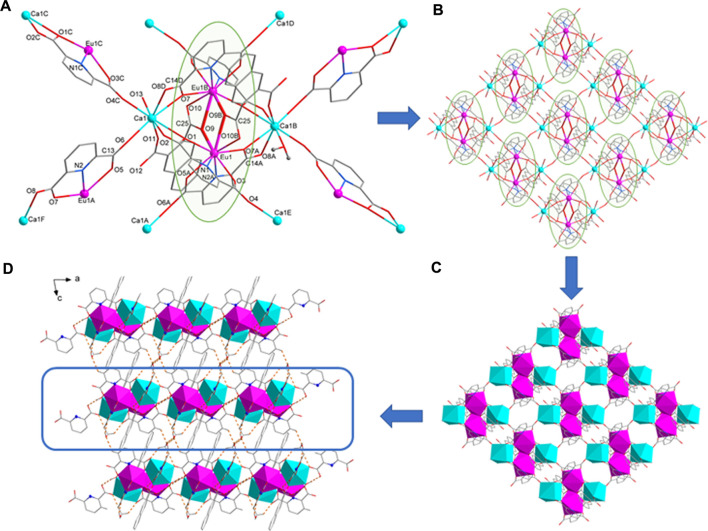
**(A)** The coordination environment of Eu1 and Ca1 ion. **(B)** 2D layer structure of **1**. **(C)** 2D layer diamond structure of **1**. **(D)** The hydrogen bond packing diagrams of **1**.

Complexes [Ln_2_ (pda)_4_(Hnda)_2_Ca_2_(H_2_O)_2_]·MeOH (Ln = Eu (**1**), Tb (**2**)) belong to triclinic system, space group $P_{\bar{1}}$ with similar crystal structure (Table 1). Taking complex **1** as an example, the structure of compound **1**-**2** is introduced. The smallest structural unit of complex **1** consists of two Eu^3+^, two Ca^2+^, four pda^2-^ ligands, two nda^2-^ ligands, two coordinated water molecules and one free methanol molecule. As shown in Figure 1A Eu1 is nine coordinated with four carboxylic acid oxygen atoms from two pda^2-^ ligands, 2 N atoms, and three carboxylic acid oxygen atoms from two nda^2-^ ligands, to form a three-capped prism structure (Supplementary Table S1). Two Eu1 atoms are connected by two O9 atoms from two nda^2-^ ligands to form a centrosymmetric binuclear [Ln_2_ (pda)_4_(Hnda)_2_]^6-^ structure. The Ca1 ion is octa-coordinated with six carboxylic acid oxygen atoms from four pda^2-^ ligands, one carboxylic acid oxygen atom from nda^2-^ ligand, and one water molecule oxygen atom, respectively. Each binuclear [Ln_2_ (pda)_4_(Hnda)_2_]^6-^ structure is bridged by six Ca(II) ions to form a two-dimensional (2D) layer structure in *ab* plane (Figures 1B,C). The methanol molecule in **1** is located between the two-dimensional layer structure and is connected to the adjacent methanol molecule *via* C27-H27B· O14K to the adjacent methanol molecule. These two methanol molecules are connected by hydrogen bonds C4-H4· O14G, C27-H27A· O4H, C27-H27C O2F linking the adjacent two-dimensional planes to form a three-dimensional supramolecular structure (Figure 1D, Supplementary Table S2).

**TABLE 1:** Crystallographic data and structural refinements for AE-Ln-CPs **1**–**6**.

Identification code	1	2	3	4	5	6
empirical formula	C_53_H_34_Ca_2_N_4_O_27_Eu_2_	C_53_H_34_Ca_2_N_4_O_27_Tb_2_	C_27_H_15_Sr_2_N_2_O_15_Eu	C_27_H_15_Sr_2_N_2_O_15_Tb	C_27_H_15_Ba_2_N_2_O_15_Eu	C_27_H_15_Ba_2_N_2_O_15_Tb
formula weight	1,540.90	1,556.84	934.61	941.57	1,034.05	1,041.01
crystal system	Triclinic	Triclinic	Monoclinic	Monoclinic	Monoclinic	Monoclinic
space group	$P_{\bar{1}}$	$P_{\bar{1}}$	P2_1_/n	P2_1_/n	P2_1_/n	P2_1_/n
a/Å	10.6235 (4)	10.6233 (5)	11.2042 (15)	11.2072 (3)	11.4694 (4)	11.4250 (4)
b/Å	10.7453 (4)	10.7427 (5)	22.694 (2)	22.7507 (5)	22.6575 (8)	22.6618 (8)
c/Å	13.4207 (5)	13.3934 (7)	11.8323 (15)	11.8641 (2)	12.2345 (4)	12.2824 (4)
α/°	68.2390 (10)	68.110 (2)	90	90	90	90
β/°	76.1470 (10)	76.032 (2)	111.564 (5)	111.6493 (8)	110.9631 (11)	111.0946 (12)
γ/°	78.4960 (10)	78.2950 (10)	90	90	90	90
volume/Å^3^	1,371.01 (9)	1,356.50 (12)	2,798.0 (6)	2,811.62 (11)	2,968.91 (18)	2,966.94 (19)
Z	1	1	4	4	4	4
ρ_cak_/g cm^−3^	1.866	1.893	2.219	2.224	2.313	2.331
M/mm^−1^	2.550	2.854	6.097	6.352	4.789	5.062
F (000)	760	766	1800	1808	1944	1952
reflection collected	18,957	18,922	14,365	43,877	34,367	30,523
unique reflections	5,357 [R (int) = 0.0236]	5,351 [R (int) = 0.0291]	6,408 [R (int) = 0.0482]	6,203 [R (int) = 0.0644]	7,301 [R (int) = 0.0329]	7,174 [R (int) = 0.0351]
goodness-of-fit on F^2^	1.021	1.070	1.031	1.035	1.078	1.023
final *R*_1_ *^a^* indexes [i ≥ 2σ(I)]	0.0213	0.0222	0.0373	0.0294	0.0232	0.0250
final *wR*_2_ *^b^* indexes [i ≥ 2σ(I)]	0.0616	0.0511	0.0892	0.0552	0.0460	0.0426
largest diff. peak and hole/e.Å^3^	0.882 and -0.312	0.803 and -0.441	1.108 and -1.163	0.947 and -1.236	0.501 and -1.077	0.656 and -0.831

a $R_{1}=\sum \left|\left|F_{0}\right|-\left|F_{c}\right|\right|/\sum \left|F_{0}\right|$ .

b $wR_{2}={\left[\sum \left[w{\left(F_{0}^{2}-F_{c}^{2}\right)}^{2}\right]/\sum \left[w{\left(F_{c}^{2}\right)}^{2}\right]\right]}^{1/2}$ .

### Structural Description of Complexes 3-6

**FIGURE 2:**
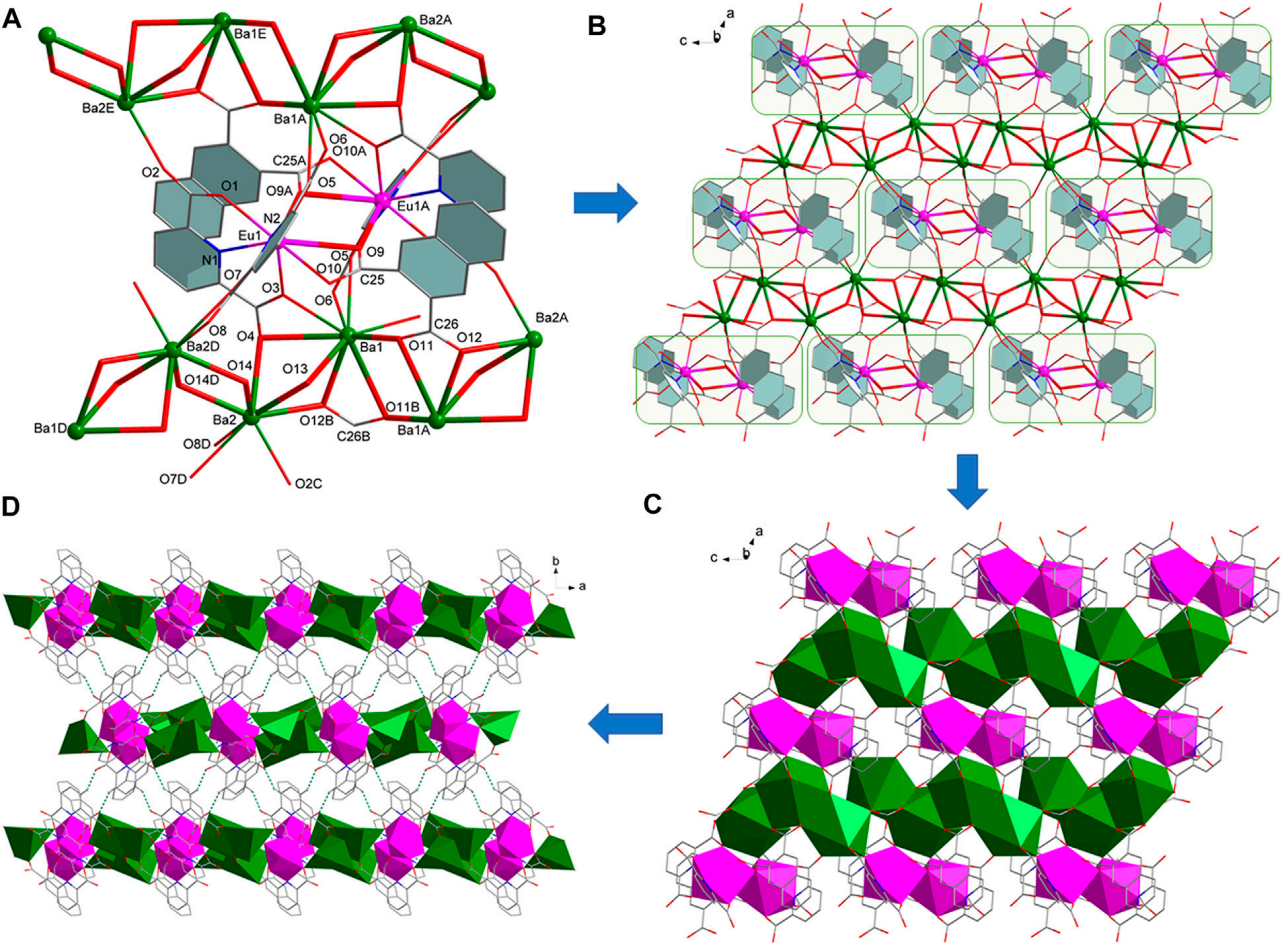
**(A)** The coordination environment of metal ions in **5**. **(B)** 2D layer structure of **5**. **(C)** 2D layer diamond structure of 5. **(D)** Hydrogen bond packing diagrams of 5.

Complexes [Ln (pda)_2_ (nda)AE_2_(HCOO)(H_2_O)] (AE = Sr, Ln = Eu (**3**), Tb (**4**); AE = Ba, Ln = Eu (**5**), Tb (**6**)) belong to monoclinic system, space group *P*2_1/*n*_, and are 2D layered bimetallic complexes with similar crystal structures (Table 1). Therefore, we take complex **5** as an example to introduce the structure of compound **3**–**5**. The smallest symmetrical unit of complex **5** consists of one Eu^3+^, two Ba^2+^, two pda^2-^ ligands, one nda^2-^ ligand, one formic acid ion and one coordinated water molecule. The coordination environment of Eu1 ion in **5** is the same as that of Eu1 in complex **1**. And two Eu1 atoms are connected by two O9 atoms from two nda^2-^ ligands to form a centrosymmetric binuclear [Eu_2_ (pda)_4_ (nda)_2_]^6-^ structure (Figure 2A). Each [Eu_2_ (pda)_4_ (nda)_2_]^6-^ structure is linked to six Ba(II) ions. There two different coordination modes of Ba(II) ion. The coordination environment of Ba1 in 5 is different from that of Ca1 in **1**. The Ba1 ion is nine-coordinated with four oxygen atoms from two pda^2-^ ligands, three O atoms from two nda^2-^ ligands, one oxygen atom from one formic acid ion and one water molecule to form a three-capped prism structure. Ba1 and Ba1 is bridged by two oxygen atoms O11 of two nda^2-^ ligands. The Ba2 ion is eight-coordinated with four oxygen atoms from three pda^2-^ ligands, one oxygen atom from one nda^2-^ ligand and three oxygen atoms from two formic acids to form a distorted dodecahedral structure (Supplementary Table S1). Ba2 is connected to Ba2 by O14 and to Ba1 by o12, o13, and o14, forming a 1D (BaO)_n_ Z-shaped chain structure extending in the direction of the *c*-axis (Figure 2B). The binuclear [Ln_2_ (pda)_4_ (nda)_2_]^6-^ structure is connected by 1D (BaO)_n_ Z-shaped chain to form a 2D layered structure (Figure 2C). Intermolecular hydrogen bonding C (9)-H (9) O (8)F and C (11)-H (11) O (6)G connects adjacent two-dimensional layer structures of **5** to form a three-dimensional supramolecular structure. The coordinated water molecule (O15) is bonded to two carboxyl oxygen atoms (O13 and O2) through intramolecular O-H O hydrogen bonds, which enhances the stability of the crystal structure (Figure 2D, Supplementary Table S2 **)**.

The structure of complex **6** is similar to complexes **3–5**. The difference lies in the way of bridging between the two Tb1 ions, see Supplementary Figure S1. The two Tb1s are bridged by two carboxyl groups, and due to the long distance of Tb1-O9, no bridging is formed through O9 as in the case of complexes **3-5**.

### PXRD and Thermal Stability Analysis

The phase purity of the as-synthesized complexes **1**-**6** were further confirmed by PXRD measurement, and each PXRD pattern of the as-synthesized samples **1**-**6** are in good agreement with the simulated ones (Supplementary Figure S2), indicating that the phase purity of complexes **1**-**6** is relatively high. To discuss the stability of complexes **1-6** in common solvents, we measured the XRD patterns of complexes **1** and **5** before and after their dispersion in ethanol, methanol, DMF, and water, and found that they were stable in all these solvents (Supplementary Figure S3). We also measured the XRD patterns of samples **1** and **5** before and after their dispersion in aqueous solutions with different pH values and found that complexes **1** and **5** were partially decomposed at pH = 1 and 2 and was more stable between pH = 3 and 11 (Supplementary Figure S4).

The thermal stability of complexes **1**-**6** was investigated at N_2_ atmosphere and the resultant plot is shown in Supplementary Figure S5. Thermal analyses of the obtained complexes **1**-**2** reveal similar features due to their isostructural nature, and the same is true for complexes **3**–**6**. The first weight loss of 4.2% (cacld. 4.4%) and 4.1% (cacld. 4.4%) of complexes **1** and **2** from room temperature to 160°C is attributed to the removal of two coordinated water molecules and a molecule of methanol. As for complexes **3**-**6**, the complexes lost a coordinated water molecule at approximately 150°C (exp. 0.5%, cacld. 1.9% for **3**; exp. 1.1%, cacld. 1.9% for **4**; exp. 0.9%, cacld. 1.7% for **5**; exp. 1.6%, cacld. 1.7% for **6**). The decomposition temperatures of all complexes exceeded 430°C, which indicates that complexes **1**-**6** have good thermal stability. The decomposition temperatures of all these complexes are higher than those of the rare earth complexes due to the coordination of alkaline earth metal ions, which reduces the ionic polarization of rare earth cations and enhances the stability of the complexes.

### IR

The IR spectra of complexes **1**-**6** were determined by KBr Tablet in the range of 4,000–400 cm^−1^. It can be seen from Supplementary Figures S6–S8 that the wide band at 3450cm^−1^ (**1**)、3,430 cm^−1^ (**2**–**6**) in the complexes **1**-**6** spectrum can be attributed to the stretching vibrations of O–H, revealed the presence of water molecules (Fan et al., 2020; Lyu et al., 2021). Besides, C-H in-plane bending vibration at 1,480, 1,430, 1,410 cm^−1^ **1**) mains the existence of ligands (1,470, 1,440, 1,410 cm^−1^ for **3**–**6**) (Yang et al., 2019). For complexes **1**-**2**, one sharp band appeared at 1720 cm^−1^ is due to the C=O absorption and C=C conjugation stretching vibrations of ester (Yang et al., 2019). For complexes **5**-**6**, two sharp bands appeared at 1,610, 1,550 cm^−1^ can be ascribed to the asymmetric stretching vibration of the C=O group from carboxylate (1,610 and 1,550 cm^−1^ for **3**–**4**) (Yang et al., 2019; Kaur et al., 2020), which indicated that ligands coordinate with metal ions. In addition, the band from 934 to 698 cm^−1^ can be attributed to the out of plane bending vibration of aromatic hydrocarbon C-H (Guo et al., 2020).

### Luminescence Properties

The solid-state photoluminescence (PL) properties of complexes **1**-**6** were studied at room temperature. As shown in Supplementary Figure S9, the emission spectra of complexes **1**, **3** and **5** are similar, showing the characteristic emission peaks of metal Eu(III) ions, and the emission peaks of complexes **2**, **4**, and **6** are similar, showing the characteristic emission peaks of metal Tb(III) ions. Now, take complexes **3** and **4** as examples to analysis the emission spectra of complexes **1–6**. For complex **3**, upon excitation at 356 nm, the two major peaks of **3** are centered at 591(594), 614(618) nm, which can be attributed to the ^5^D_0_→^7^F_J_ (J = 1,2) transition of Eu^3+^, respectively. The highest intensity emission peaks of complexes **1**, **3**, and **5** all appear at 614 nm, indicating that they all emit red light. In the same light, upon excitation at 347 nm, the four major peaks of **4** are centered at 490, 545, 593, and 618 nm, which can be attributed to the ^5^D_4_→^7^F_J_ (J = 6,5,4,3) transitions of Tb^3+^, respectively. The most intense emission is centered at 545 nm, indicating that complex **4** emits green light. For the remaining complexes, the main intense emission bands were observed at λ_em_ = 618 nm (λ_ex_ = 354 nm) for **1**, 545 and 615 (618) nm (λ_ex_ = 323 nm) for **2**, 614 nm (λ_ex_ = 356 nm) for **3**, 546 nm (λ_ex_ = 347 nm) for **4**, 618 nm (λ_ex_ = 342 nm) for **5** and 547 nm (λ_ex_ = 345 nm) for **6**. Notably, unlike complexes **4** and **6**, the maximum emission peak for complex **2** occurs at 545 and 615 (618) nm, indicating that complex **2** emits lime-green light. This is consistent with the color of the emission light observed on the test paper for **1-6** under 254 nm UV light (Supplementary Figure S10).

As shown in Supplementary Figure S11 and Supplementary Table S3, quantum yields (QYs) of AE-Eu(III) complexes **1**, **3** and **5** are *Ф*_total_ = 63.01, 60.61 and 87.39%, respectively. The fluorescence lifetimes of complexes **1**, **3** and **5** are τ_obs_ = 1930.94 ± 2.19, τ_obs_ = 2049.48 ± 2.21 and τ_obs_ = 2,413.04 ± 2.39 µs, respectively, and are single exponential decay. The quantum yields of **2**, **4** and **6** were *Ф*_total_ = 4.61% (**2**), 0.65% **4**) and 5.56% (**6**), respectively, and the fluorescence lifetimes are τ_obs_ = 145.86 ± 0.95 µs (**2**), 109.24 ± 0.69 µs **4**) and 208.95 ± 1.46 µs (**6**), respectively, with double exponential decay. It can be seen that the fluorescence lifetime and quantum yield of complexes **2**, **4** and **6** are much lower than those of complexes **1**, **3** and **5**. In comparison with the previously reported AE-Ln bimetallic complexes with H_2_pda as ligand by our group, it was found that the fluorescence lifetime of AE-Eu-CPs increased with the addition of H_2_nda, regardless of the structure, and on the contrary the fluorescence lifetime of AE-Tb-CPs decreased (Chen et al., 2013; Chen et al., 2019; Chen et al., 2020). This may because the conjugation effect of H_2_nda matches the energy of Eu(III) ions and can act as an antenna to sensitize the luminescence of Eu(III) ions, but it does not match the energy of Tb(III) ion, so it weakens the luminescence of Tb(III) ions. This is consistent with the weaker luminescence of the complexes of Tb-nda reported in the literature (Hu et al., 2015). By comparing the fluorescence properties of complexes **1-6**, it was found that complexes **5** and **6** had the highest fluorescence lifetime and quantum yield in complexes AE-Eu-CPs and AE-Tb-CPs, respectively. This suggests that the one-dimensional (BaO)_n_ chains may facilitate energy transfer.

### Sensing of Metal Cations

The intense luminescence of complexes **1**-**6** promotes us to investigate its ability to sense common metal cations. The finely ground samples of complexes **1**–**6** (2 mg) were dispersed in the aqueous solutions of MCl_n_ (10 ml, 1 × 10^–2^ mol L^−1^, M^n+^ = Fe^3+^, Cu^2+^, Cd^2+^, Mg^2+^, Ni^2+^, Co^2+^, Ca^2+^, Ba^2+^, Sr^2+^, Li^+^, Na^+^, K^+^, Al^3+^, Fe^2+^, Pb^2+^, Cr^3+^, Mn^2+^ and Zn^2+^, respectively.) to form a suspension at room temperature. The mixtures were used for luminescence measurements, and the luminescence data were collected and compared at the room temperature. The results revealed that various metal ions display markedly different effects on the luminescence of the complexes.

**FIGURE 3:**
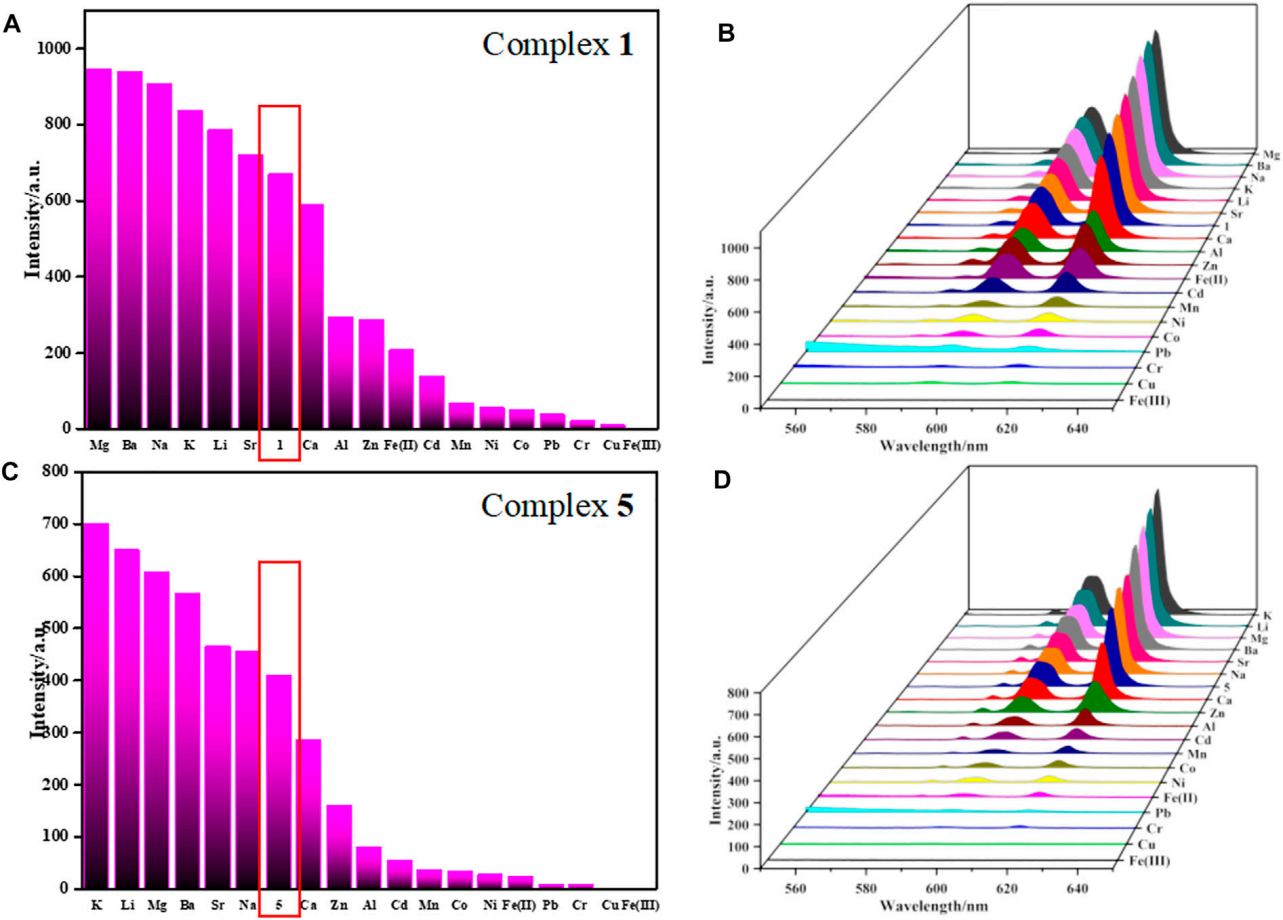
**(A,C)** are bar graph of FL response of **1** and **5** towards different metal ions (λ_max_ = 616 nm), respectively. **(B,D)** are 3D plane of luminescent intensities of **1** and **5** in aqueous solution with various metal ions, respectively.

As shown in Figure 3, Supplementary Figures S12–S16 **,** the corresponding luminescence curves still show characteristic emission peaks of Eu^3+^ and Tb^3+^ ions, and only the highest emission peak around 616 nm or 546 nm was monitored, respectively. The results reveal that the luminescence intensities of **1-6** have a certain degree response for all metal ions, but the responsiveness is dependent on the species of metal ions. Taking complexes **1** and **5** as examples to introduce their sensing of metal ions because their difference in structure. For complex **1**, Mg^2+^, Ba^2+^, Na^+^, K^+^, Li^+^, Sr^2+^ have different degrees of fluorescence enhancement on **1**; Ca^2+^, Al^3+^, Zn^2+^, Cd^2+^, Mn^2+^, Ni^2+^, Co^2+^, Fe^2+^, Pb^2+^ and Cr^3+^ show negligible effects on its fluorescence; Fe^3+^ and Cu^2+^ exhibit a moderate degree of quenching (Figures 3A,B), whereas Fe^3+^ gives the most significant quenching with the efficiency as high as 99%. The quenching efficiency Q was calculated by the following formula:

$$\text{Q}=\left({\text{I}}_{\text{0}}=\text{I}\right)/{\text{I}}_{0}\times 100\%$$ (1)

I_0_ and I are the maximum luminescence intensity before and after adding the target. As for **5**, Sr^2+^ and Na^+^ have negligible effect on the fluorescence of **5**, K^+^, Li^+^, Mg^2+^, Ba^2+^, Ca^2+^, Zn^2+^, Al^3+^, Cd^2+^, Mn^2+^, Co^2+^, Ni^2+^, Fe^2+^, Pb^2+^ and Cr^3+^ exhibit a moderate (or observed) degree of responding, but Cu^2+^ and Fe^3+^ represent the most significant quenching effect based on **5**, especially for Fe(III) ions (Figures 3C,D). The Q of complex **5** toward Fe^3+^ was evaluated to be 99%, same as complex **1**. The color of complexes **1** and **5** suspensions changed from red to dark under ultraviolet light, obviously, complexes **1** and **5** can highly efficiently sense Fe^3+^ ions by fluorescent quenching, which can also be easily recognized by naked eye (Supplementary Figure S17).

**FIGURE 4:**
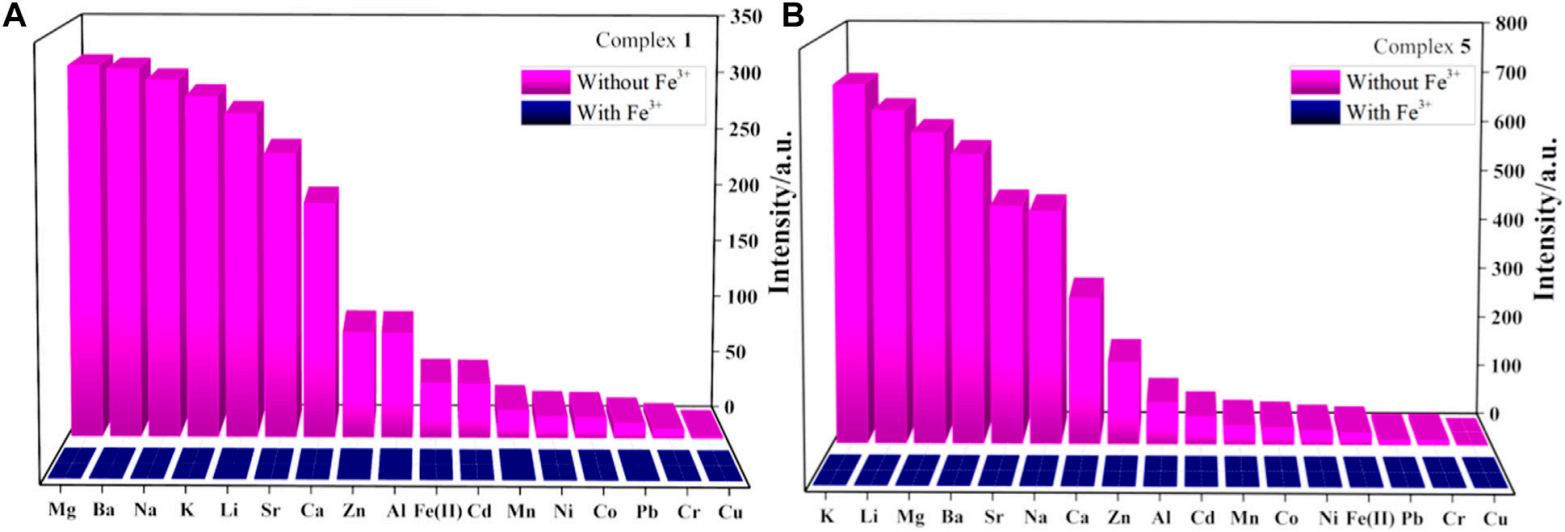
**(A,B)** are fluorescence intensity of complexes **1** and **5** dispersed in aqueous solutions of mixed cations without or with Fe^3+^, respectively.

It should be noted that usually many metal ions coexist in actual biological and environmental systems. To investigate the detection selectivity of complexes **1**-**6** for Fe^3+^ and the influence of other metal ions on the detection of Fe^3+^, anti-interference experiments were carried out to verify the high selectivity of complexes **1**-**6** to Fe^3+^ detection (Figure 4, Supplementary Figures S18–S21). As shown in Figure 4, once Fe^3+^ ions are introduced into the aqueous solution of complexes **1** or **5** and other metal cations (1 × 10^–2^ M), the luminescence is quenched obviously. The result reveals that the interference from other metal cations (Cu^2+^, Cd^2+^, Mg^2+^, Ni^2+^, Co^2+^, Ca^2+^, Ba^2+^, Sr^2+^, Li^+^, Na^+^, K^+^, Al^3+^, Fe^2+^, Pb^2+^, Cr^3+^, Mn^2+^ and Zn^2+^) can be neglected and further confirms the selectivity of complexes **1-6** for Fe^3+^ detection.

**FIGURE 5:**
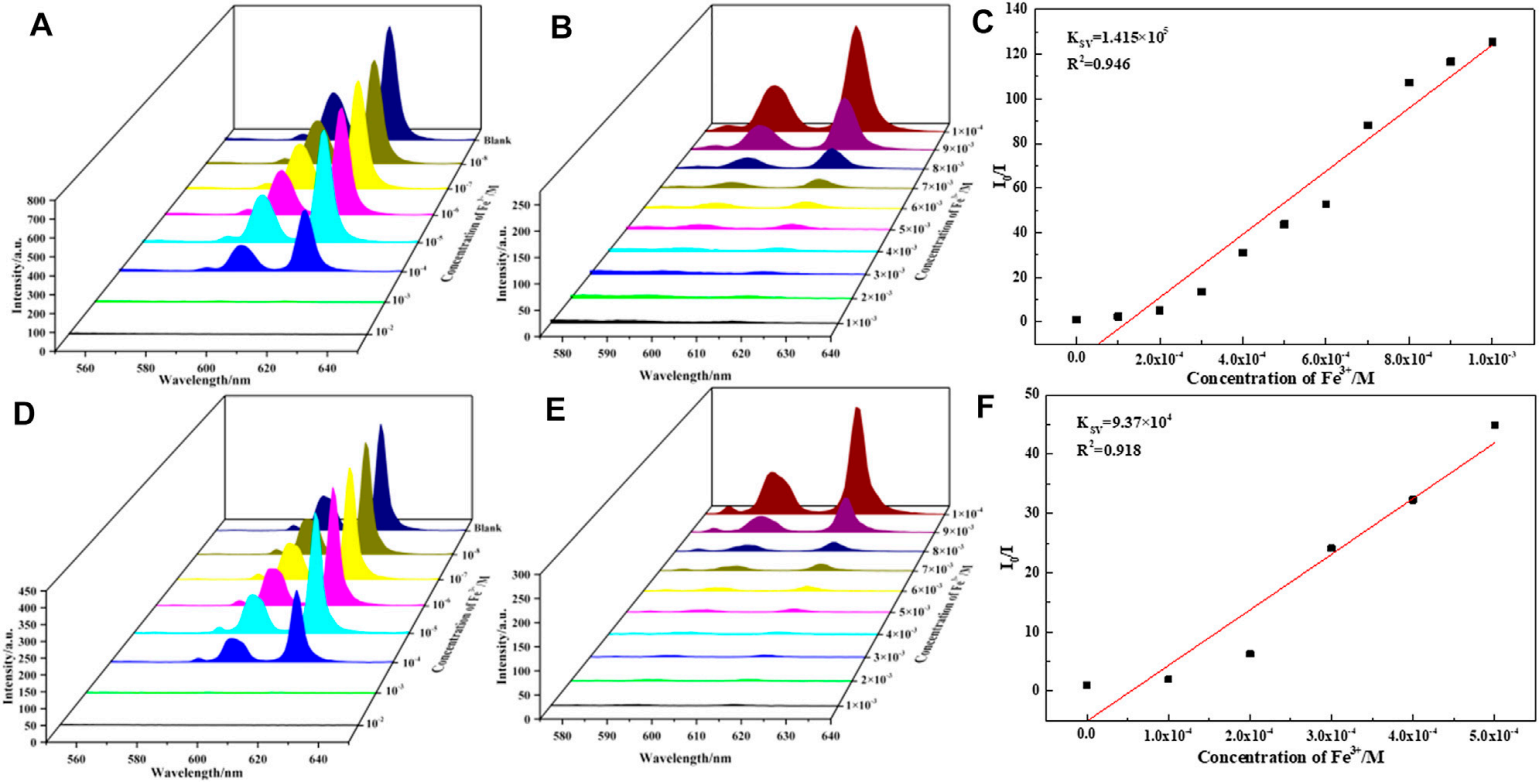
**(A,D)** are luminescence spectra of **1** and **5** dispersed in aqueous solutions of FeCl_3_ (10^−2^–10^−8^ M, 10 ml H_2_O, 2 mg **1** and **5**), respectively. **(B,E)** are luminescence spectra of **1** and **5** dispersed in aqueous solutions of FeCl_3_ (10^−3^–10^−4^ M, 10 ml H_2_O, 2 mg **1** and **5**), respectively. **(C,F)** are linear dependence between the quenching efficiency and the concentration of Fe^3+^ in the range of 0.1–1 mM, respectively.

To evaluate the sensing sensitivity of complexes **1-6**, quantitative fluorescence titration experiments were carried out in the luminescence intensities of complexes **1**-**6** in presence of Fe(III) ions (Figure 5, Supplementary Figures S22–S25). It was found that the luminescence intensity of Fe-incorporated **1**–**6** has a great relationship with the concentration of the metal ions. As shown in Figures 5A,D, the luminescence intensity of the complexes **1** and **5** decreased drastically and monotonically when the Fe^3+^ concentration increased from 1 × 10^–2^ to 1 × 10^–8^ M, and it is worth noting that when the concentration of Fe^3+^ ion rises from 10^–4^ to 10^–3^ M, the fluorescence intensity of complexes **1** and **5** drops sharply, so we refine the concentration from 10^–4^ to 10^–3^ M (Figures 5B,E). The relationship between the luminescence intensity of complexes **1**-**6** and the concentration of Fe^3+^ conform to the Stern–Volmer equation:

$${\text{I}}_{\text{0}}/\text{I}=1+K_{\text{SV}}\left[\text{M}\right]$$ (2)

where I_0_ and I are the luminescence intensity of complexes **1** and **5** before and after adding Fe^3+^, respectively, [M] is the concentration of Fe^3+^. *K*_SV_ is the quenching constant, the value of complexes **1** and **5** are calculated as 1.41 × 10^5^ and 9.37 × 10^4^, respectively (Figures 5C,F), which indicates a strong quenching effect on the complexes **1** and **5** luminescence. The linear correlation coefficient (R) in the Stern–Volmer curve of complexes **1** and **5** with Fe^3+^ is 0.945 and 0.918, respectively. The calculated *K*_SV_ values of complexes **2**, **3**, **4**, and **6** are 7.10 × 10^4^ M^−1^ (for **2**), 1.70 × 10^5^ M^−1^ (for **3**), 1.57 × 10^5^ M^−1^ (for **4**), 1.27 × 10^5^ M^−1^ (for **6**), respectively (Supplementary Figures S22–S25). It is further revealed that complexes **1-6** have a high sensitivity for detecting Fe^3+^ ions in aqueous solution.

### Luminescence Quenching Mechanism

To explain a possible sensing mechanism of **1-6** toward Fe^3+^, the luminescence quenching effects were studied further. Usually, the causes of the quenching effect can be summarized as follows: 1) the collapse of framework structure; 2) cations exchange between lanthanide Eu/Tb central ions and target cations; 3) competitive absorption between the metal ions and Eu/Tb metal organic framework (Jia et al., 2020). We investigated the luminescence quenching mechanism of sensing Fe^3+^ using complex **1** and **5** as examples. First, suspensions of complexes **1** or **5** were made in aqueous solutions of Fe^3+^, and the recovered solids were subsequently subjected to powder X-ray diffraction analysis. The results obtained (Supplementary Figure S26) showed that the structures of both complexes **1** and **5** were preserved; therefore, the observed emission quenching was not related to the decomposition of complexes **1** or **5**. Second, the solids of complexes **1** and **5**, suspended after Fe^3+^ solution, were tested for solid fluorescence after repeated rinsing (Supplementary Figure S27). The curve of solid fluorescence was found to be the same as the emission peak before suspension, which indicates that no replacement of the central metal ion between the complexes and Fe^3+^ has occurred. Therefore, the fluorescence quenching mechanism can be attributed to the weak Fe^3+^-O interactions, which probably come from oxygen atoms of uncoordinated carboxyl oxygen atoms or coordinated water molecules inside the complexes (Zhang et al., 2018). To verify the above speculation, the XPS (X-ray photoelectron spectroscopy) and the UV/vis studies were performed.

For complex **1** and **5**, before and after the treatment with Fe^3+^, the XPS measurements were carried out on the Fe2P peaks (Supplementary Figure S28). It was found that the peaks of Fe 2p were absent in complexes **1** and **5**, while the samples treated with Fe^3+^ contained obvious peaks of Fe 2p. The XPS peaks of Fe2p_3/2_ of Fe^3+^ treated **1** and **5** were 712.4 and 711.5 eV. This is close to the Fe2p_3/2_ value of FeCl_3_ reported in the literature (Yamashita and Hayes, 2008). And the I1/I2 value of It indicated that the weak interactions of Fe^3+^-O were existed. The ratio of the signal intensity at the high binding energy end (I_1_) to that of the Fe2p_3/2_ peak (I_2_) is 0.74 and 0.74, respectively, which is greater than 0.65 and is close to the I_1_/I_2_ value of Fe^3+^ reported in the literature (Liu and Chen, 2001). Therefore, the energy spectral signal of Fe^3+^ observed in the XPS fully indicates the existence of the weak Fe^3+^-O interactions. To further prove the above hypothesis, the UV/vis spectra were measured. After treatment of **1** and **5** with Fe^3+^, a slight shift in the solid-state UV/vis spectra appears (Supplementary Figure S29), indicating a weak interaction of 1 and 5 with Fe^3+^ ions. In addition, we also found that after the treatment with Fe^3+^, the color of complexes **1** and **5** is slightly yellowish (Supplementary Figure S30), while both complexes 1 and 5 are white crystal. When the samples were washed with water several times, the color of the samples could change back to white. Therefore, the fluorescence quenching mechanism attributed to the weak Fe^3+^-O interactions between Fe^3+^ and AE-Ln-CPs.

To simplify the calculation, we simply calculated the HOMO and LUMO orbital energies of monomers and dimers of complexes **1** and **5** (Supplementary Table S4). The HOMO-LUMO gap is different from the excitation energy, but it can be used only as an approximation. The energy gap of monomers of complexes **1** and **5** is 0.4 eV, but the dimer is only 0.2 eV. We obtain that 1) the effect of the packing configurations is obvious and 2) crystal compounds are easy to return to the ground state through nonradiative transition from the excited state due to the aggregation effect (Supplementary Figure S31). This aggregation effect is affected when the iron ions form weak interactions with the complexes, so that complexes **1** and **5** exhibit effective fluorescence quenching.

## Conclusion

Six new heterometallic dual-liganded AE-Ln-CPs were synthesized by solvothermal method. These complexes exhibit two different two-dimensional structures, based on different coordination modes of alkaline earth metal ions. All of them show good thermal stability blow 430°C. Their solid-state fluorescence all exhibits the characteristic emission spectra of rare-earth ions. Interestingly, the fluorescence lifetimes and quantum yield of AE-Eu-CPs (**1**, **3**, **5**) are higher, while those of AE-Tb-CPs (**2**, **4**, **6**) are lower. The analysis revealed that the energy match of 2,3-naphthalenedicarboxylic acid with Eu(III) could sensitize the luminescence of Eu(III) ion, but not with Tb(III), so the fluorescence of AE-Tb-CPs was weak. And comparing the effect of three different alkaline earth metal ions on fluorescence, it was found that the formation of (BaO)_n_ chains had a more obvious effect on fluorescence enhancement. The identification of metal ions in aqueous solution was done for six complexes, and it was found that they all have a keen selective sensing effect on Fe^3+^ and are not affected by other metal ions, such as Cu^2+^, Cd^2+^, Mg^2+^, Ni^2+^, Co^2+^, Ca^2+^, Ba^2+^, Sr^2+^, Li^+^, Na^+^, K^+^, Al^3+^, Fe^2+^, Pb^2+^, Cr^3+^, Mn^2+^ and Zn^2+^. By studying the XRD, XPS, UV/Vis of the samples before and after treatment with Fe^3+^, a possible mechanism of selective fluorescence sensing is proposed: Fe^3+^ ions and AE-Ln-CPs form a weak Fe^3+^-O interaction. The sensitive recognition of Fe^3+^ by these complexes is expected to be applied to Fe^3+^ ion sensing in the environment and living organisms.

## Data Availability

The original contributions presented in the study are publicly available. This data can be found here: https://www.ccdc.cam.ac.uk/, 2145453-2145458.
