# Supplementary material for: Heterometallic Dual-Liganded AE-Ln-CPs Luminescent Probes for Efficient Sensing of Fe(III) Ions
Source: Front Chem. 2022 Apr 7;10:865447. doi: 10.3389/fchem.2022.865447 (PMC9021488; doi:10.3389/fchem.2022.865447)
Supplement: Supplementary file 1 [file DataSheet1.docx]

Supplementary Material

**Heterometallic Dual-liganded AE-Ln-CPs Luminescent Probes for Efficient Sensing of Fe(III) Ions**

**Jieqiong Hou^1,2^, Yanmei Chen^1^*, Shuixiang Zou^1^, Wenwen Dong^2^, Zhenghua Ju^3^, Junqi Lin^1^, Zhijun Ruan^1^, Shanshan Liu^1^, Zhengfang Tian^1^****

^1^Hubei Key Laboratory for Processing and Application of Catalytic Materials, College of Chemistry and Chemical Engineering, Huanggang Normal University, Huanggang, 438000, China.

^2^College of Materials and Chemical Engineering, Key Laboratory of Inorganic Nonmetallic Crystalline and Energy Conversion Materials, Hubei Provincial Collaborative Innovation Center for New Energy Microgrid, China Three Gorges University, Yichang, Peoples Republic of China.

^3^Analysis and Testing Center, Lanzhou University, Lanzhou, 730000, China

*** Correspondence:**

Yanmei Chen: cingym@163.com

Zhengfang Tian: tzf7801@163.com

# 1. IR data and Element Analysis

[Eu_2_(pda)_4_(nda)_2_Ca_2_(MeOH)(H_2_O)_2_] (**1)**: IR (KBr, cm^-1^): 3650 (w), 1720 (s), 1630 (s), 1570 (s), 1480 (s), 1430 (m), 1410 (s), 1280 (m), 1190 (m), 1070 (m), 1020 (m), 932 (m), 857 (s), 815 (s), 772 (s), 738 (s), 695 (s), 665 (m), 609 (m), 474 (m), 429 (s). Anal. Calcd. (%) for C_53_H_34_Ca_2_N_4_O_27_Eu_2_: C, 41.26; H, 2.22; N, 3.63. Found (%): C, 41.22; H, 2.32; N, 3.28.

[Tb_2_(pda)_4_(nda)_2_Ca_2_(MeOH)(H_2_O)_2_] (**2)**: IR (KBr, cm^-1^): 3430 (s), 1720 (s), 1630 (s), 1570 (m), 1480 (s), 1430 (s), 1410 (s), 1280 (m), 1190 (m), 1070 (m), 1020 (m), 934 (m), 859 (m), 814 (m), 772 (s), 740 (s), 696 (m), 665 (m), 609 (m), 475 (w), 432 (m). Anal. Calcd. (%) for C_53_H_34_Ca_2_N_4_O_27_Tb_2_: C, 40.89; H, 2.20; N, 3.60. Found (%): C, 40.82; H, 2.22; N, 3.57.

[Eu(pda)_2_(nda)Sr_2_(HCOO)(H_2_O)] (**3)**: IR (KBr, cm^-1^): 3430 (s), 1650 (s), 1610 (s), 1550 (s), 1470 (s), 1440 (s), 1410 (s), 1380 (w), 1350 (s), 1280 (s), 1190 (m), 1150 (m), 1070 (m), 1020 (m), 925 (m), 865 (m), 820 (m), 787 (s), 744 (s), 700 (m), 663 (m), 610 (m), 472 (m), 434 (m). Anal. Calcd. (%) for C_27_H_15_Sr_2_N_2_O_15_Eu: C, 34.70; H, 1.62; N, 3.00. Found (%): C, 34.68; H, 1.70; N, 2.98.

[Tb(pda)_2_(nda)Sr_2_(HCOO)(H_2_O)] (**4):** IR (KBr, cm^-1^): 3430 (w), 1650 (s), 1610 (s), 1550 (s), 1470 (m), 1440 (s), 1410 (m), 1380 (m), 1350 (m), 1280 (m), 1190 (w), 1150 (w), 1070 (m), 1020 (m), 927 (m), 865 (m), 818 (m), 786 (s), 739 (s), 699 (m), 662 (w), 610 (w), 472 (w), 435 (m). Anal. Calcd. (%) for C_27_H_15_Sr_2_N_2_O_15_Tb: C, 34.44; H, 1.61; N, 2.98. Found (%): C, 34.57; H, 1.65; N, 2.88.

[Eu(pda)_2_(nda)Ba_2_(HCOO)(H_2_O)] (**5):** IR (KBr, cm^-1^): 3430 (s), 1670 (m), 1640 (s), 1610 (s), 1540 (s), 1470 (m), 1440 (s), 1410 (m), 1380 (m), 1350 (m), 1280 (s), 1200 (m), 1080 (m), 1020 (m), 925 (s), 862 (s), 817 (s), 785 (s), 740 (s), 698 (s), 663 (m), 553 (m), 473 (m), 432 (s). Anal. Calcd. (%) for C_27_H_15_Ba_2_N_2_O_15_Eu: C, 31.36; H, 1.46; N, 2.71. Found (%): C, 31.23; H, 1.53; N, 2.75.

[Tb(pda)_2_(nda)Ba_2_(HCOO)(H_2_O)] (**6):** IR (KBr, cm^-1^): 3430 (w), 1670 (m), 1640 (s), 1610 (s), 1540 (s), 1470 (m), 1440 (s), 1410 (m), 1380 (m), 1350 (w), 1280 (m), 1200 (w), 1080 (w), 1020 (m), 926 (m), 865 (m), 814 (m), 785 (m), 741 (s), 698 (m), 663 (w), 534 (w), 473 (w), 434 (m). Anal. Calcd. (%) for C_27_H_15_Ba_2_N_2_O_15_Tb: C, 31.15; H, 1.45; N, 2.69. Found (%): C, 31.03; H, 1.55; N, 2.57.

# 2. Tables and figures

**Table S1.** Selected Bond Lengths (Å) and Bond Angles (°) for Complexes **1**-**6.**

| Complex **1** Bond | Dist. | Bond | Dist. |
| --- | --- | --- | --- |
| Eu(1)-O(9) | 2.3820(19) | Ca(1)-O(6) | 2.291(2) |
| Eu(1)-O(3) | 2.402(2) | Ca(1)-O(11) | 2.373(2) |
| Eu(1)-O(7)A | 2.4214(19) | Ca(1)-O(4)C | 2.420(2) |
| Eu(1)-O(1) | 2.448(2) | Ca(1)-O(13) | 2.430(3) |
| Eu(1)-O(5)A | 2.470(2) | Ca(1)-O(8)D | 2.488(2) |
| Eu(1)-O(10)B | 2.517(2) | Ca(1)-O(7)D | 2.5551(19) |
| Eu(1)-N(1) | 2.518(2) | Ca(1)-O(1) | 2.570(2) |
| Eu(1)-N(2)A | 2.548(2) | Ca(1)-O(2) | 2.640(2) |
| Eu(1)-O(9)B | 2.628(2) |  |  |
| Angle | (**°**) | Angle | (**°**) |
| O(9)-Eu(1)-O(3) | 153.38(7) | O(6)-Ca(1)-O(11) | 81.15(8) |
| O(9)-Eu(1)-O(7)A | 78.93(7) | O(6)-Ca(1)-O(4)C | 76.07(9) |
| O(3)-Eu(1)-O(7)A | 78.47(7) | O(11)-Ca(1)-O(4)C | 133.33(8) |
| O(9)-Eu(1)-O(1) | 78.62(7) | O(6)-Ca(1)-O(13) | 108.02(11) |
| O(3)-Eu(1)-O(1) | 127.15(7) | O(11)-Ca(1)-O(13) | 72.48(10) |
| O(7)A-Eu(1)-O(1) | 150.65(7) | O(4)C-Ca(1)-O(13) | 76.70(8) |
| O(9)-Eu(1)-O(5)A | 95.85(7) | O(6)-Ca(1)-O(8)D | 133.61(8) |
| O(3)-Eu(1)-O(5)A | 86.27(8) | O(11)-Ca(1)-O(8)D | 144.46(7) |
| O(7)A-Eu(1)-O(5)A | 126.12(7) | O(4)C-Ca(1)-O(8)D | 73.17(8) |
| O(1)-Eu(1)-O(5)A | 74.93(7) | O(13)-Ca(1)-O(8)D | 97.71(10) |
| O(9)-Eu(1)-O(10)B | 114.58(7) | O(6)-Ca(1)-O(7)D | 172.24(8) |
| O(3)-Eu(1)-O(10)B | 74.13(7) | O(11)-Ca(1)-O(7)D | 92.75(7) |
| O(7)A-Eu(1)-O(10)B | 78.39(7) | O(4)C-Ca(1)-O(7)D | 111.69(8) |
| O(1)-Eu(1)-O(10)B | 94.12(8) | O(13)-Ca(1)-O(7)D | 74.36(9) |
| O(5)A-Eu(1)-O(10)B | 145.19(7) | O(8)D-Ca(1)-O(7)D | 51.92(7) |
| O(9)-Eu(1)-N(1) | 142.06(7) | O(6)-Ca(1)-O(1) | 102.03(8) |
| O(3)-Eu(1)-N(1) | 64.06(7) | O(11)-Ca(1)-O(1) | 74.37(7) |
| O(7)A-Eu(1)-N(1) | 136.31(7) | O(4)C-Ca(1)-O(1) | 149.97(8) |
| O(1)-Eu(1)-N(1) | 63.45(7) | O(13)-Ca(1)-O(1) | 130.42(8) |
| O(5)A-Eu(1)-N(1) | 74.94(7) | O(8)D-Ca(1)-O(1) | 88.57(8) |
| O(10)B-Eu(1)-N(1) | 70.67(7) | O(7)D-Ca(1)-O(1) | 71.53(7) |
| O(9)-Eu(1)-N(2)A | 82.10(7) | O(6)-Ca(1)-O(2) | 78.65(9) |
| O(3)-Eu(1)-N(2)A | 75.38(7) | O(11)-Ca(1)-O(2) | 113.83(8) |
| O(7)A-Eu(1)-N(2)A； | 63.70(7) | O(4)C-Ca(1)-O(2) | 100.91(7) |
| O(1)-Eu(1)-N(2)A | 130.72(7) | O(13)-Ca(1)-O(2) | 171.84(10) |
| O(5)A-Eu(1)-N(2)A | 62.47(7) | O(8)D-Ca(1)-O(2) | 74.14(8) |
| O(10)B-Eu(1)-N(2)A | 135.06(8) | O(7)D-Ca(1)-O(2) | 99.66(7) |
| N(1)-Eu(1)-N(2)A | 122.12(7) | O(1)-Ca(1)-O(2) | 50.27(6) |
| O(9)-Eu(1)-O(9)B | 65.25(7) | O(5)A-Eu(1)-O(9)B | 148.36(7) |
| O(3)-Eu(1)-O(9)B | 122.28(7) | O(10)B-Eu(1)-O(9)B | 50.12(6) |
| O(7)A-Eu(1)-O(9)B | 77.08(6) | N(1)-Eu(1)-O(9)B | 103.82(7) |
| O(1)-Eu(1)-O(9)B | 76.47(7) | N(2)A-Eu(1)-O(9)B | 133.06(7) |
| Complex **2** Bond | Dist. | Bond | Dist. |
| Tb(1)-O(9) | 2.3471(19) | Ca(1)-O(1) | 2.5726(19) |
| Tb(1)-O(3) | 2.3798(19) | Ca(1)-O(2) | 2.618(2) |
| Tb(1)-O(5)A | 2.4009(19) | Ca(1)-O(11) | 2.380(2) |
| Tb(1)-O(1) | 2.4237(19) | Ca(1)-O(8)C | 2.301(2) |
| Tb(1)-O(7)A | 2.4524(19) | Ca(1)-O(13) | 2.449(2) |
| Tb(1)-O(10)A | 2.476(2) | Ca(1)-O(6) | 2.469(2) |
| Tb(1)-N(1) | 2.492(2) | Ca(1)-O(5) | 2.5489(19) |
| Tb(1)-N(2)A | 2.519(2) | Ca(1)B-O(4) | 2.429(2) |
| Tb(1)-O(9)A | 2.664(2) |  |  |
| Angle | (**°**) | Angle | (**°**) |
| O(9)-Tb(1)-O(3) | 152.62(7) | O(8)C-Ca(1)-O(11) | 80.22(8) |
| O(9)-Tb(1)-O(5)A | 79.12(7) | O(8)C-Ca(1)-O(4)D | 75.47(8) |
| O(3)-Tb(1)-O(5)A | 77.78(7) | O(11)-Ca(1)-O(4)D | 132.12(8) |
| O(9)-Tb(1)-O(1) | 78.19(7) | O(8)C-Ca(1)-O(13) | 106.75(9) |
| O(3)-Tb(1)-O(1) | 128.35(7) | O(11)-Ca(1)-O(13) | 72.05(9) |
| O(5)A-Tb(1)-O(1) | 149.88(7) | O(4)D-Ca(1)-O(13) | 76.41(8) |
| O(9)-Tb(1)-O(7)A | 95.55(7) | O(8)C-Ca(1)-O(6) | 134.60(8) |
| O(3)-Tb(1)-O(7)A | 86.85(7) | O(11)-Ca(1)-O(6) | 144.71(7) |
| O(5)A-Tb(1)-O(7)A | 127.11(6) | O(4)D-Ca(1)-O(6) | 73.59(8) |
| O(1)-Tb(1)-O(7)A | 74.80(7) | O(13)-Ca(1)-O(6) | 97.31(9) |
| O(9)-Tb(1)-O(10)A | 114.53(7) | O(8)C-Ca(1)-O(5) | 171.84(8) |
| O(3)-Tb(1)-O(10)A | 74.99(7) | O(11)-Ca(1)-O(5) | 92.56(7) |
| O(5)A-Tb(1)-O(10)A | 78.87(7) | O(4)D-Ca(1)-O(5) | 112.46(7) |
| O(1)-Tb(1)-O(10)A | 92.86(7) | O(13)-Ca(1)-O(5) | 74.25(8) |
| O(7)A-Tb(1)-O(10)A | 144.64(7) | O(6)-Ca(1)-O(5) | 52.27(6) |
| O(9)-Tb(1)-N(1) | 142.11(7) | O(8)C-Ca(1)-O(1) | 102.94(8) |
| O(3)-Tb(1)-N(1) | 64.71(7) | O(11)-Ca(1)-O(1) | 74.84(7) |
| O(5)A-Tb(1)-N(1) | 136.13(7) | O(4)D-Ca(1)-O(1) | 150.62(7) |
| O(1)-Tb(1)-N(1) | 63.93(7) | O(13)-Ca(1)-O(1) | 130.24(8) |
| O(7)A-Tb(1)-N(1) | 74.45(7) | O(6)-Ca(1)-O(1) | 88.92(7) |
| O(10)A-Tb(1)-N(1) | 70.39(7) | O(5)-Ca(1)-O(1) | 71.24(6) |
| O(9)-Tb(1)-N(2)A | 81.77(7) | O(8)C-Ca(1)-O(2) | 79.57(8) |
| O(3)-Tb(1)-N(2)A | 75.06(7) | O(11)-Ca(1)-O(2) | 114.09(7) |
| O(5)A-Tb(1)-N(2)A | 64.15(7) | O(4)D-Ca(1)-O(2) | 101.29(7) |
| O(1)-Tb(1)-N(2)A | 130.82(7) | O(13)-Ca(1)-O(2) | 172.21(8) |
| O(7)A-Tb(1)-N(2)A | 63.02(7) | O(6)-Ca(1)-O(2) | 74.91(8) |
| O(10)A-Tb(1)-N(2)A | 136.26(8) | O(5)-Ca(1)-O(2) | 100.17(7) |
| N(1)-Tb(1)-N(2)A | 122.04(7) | O(1)-Ca(1)-O(2) | 50.51(6) |
| O(9)-Tb(1)-O(9)A | 65.10(7) | O(7)A-Tb(1)-O(9)A | 147.76(6) |
| O(3)-Tb(1)-O(9)A | 122.48(7) | O(10)A-Tb(1)-O(9)A | 50.01(6) |
| O(5)A-Tb(1)-O(9)A | 76.58(6) | N(1)-Tb(1)-O(9)A | 104.40(7) |
| O(1)-Tb(1)-O(9)A | 76.03(6) | N(2)A-Tb(1)-O(9)A | 132.63(7) |
| Complex **3** Bond | Dist. | Bond | Dist. |
| Eu(1)-O(9)A | 2.388(3) | Sr(1)-O(11) | 2.513(3) |
| Eu(1)-O(5) | 2.390(3) | Sr(1)-O(15) | 2.547(3) |
| Eu(1)-O(3) | 2.400(3) | Sr(1)-O(12)B | 2.558(3) |
| Eu(1)-O(1) | 2.403(3) | Sr(1)-O(3) | 2.594(3) |
| Eu(1)-O(7) | 2.433(3) | Sr(1)-O(13) | 2.605(3) |
| Eu(1)-O(10) | 2.504(3) | Sr(1)-O(5)A | 2.614(3) |
| Eu(1)-N(1) | 2.528(4) | Sr(1)-O(6)A | 2.829(3) |
| Eu(1)-N(2) | 2.534(3) | Sr(1)-O(4) | 2.905(3) |
| Eu(1)-O(9) | 2.672(4) | Sr(1)-O(11)B | 3.092(3) |
| Sr(2)-O(2)C | 2.462(3) | Sr(2)-O(12)B | 2.642(3) |
| Sr(2)-O(4) | 2.530(3) | Sr(2)-O(13) | 2.668(3) |
| Sr(2)-O(14)D | 2.574(3) | Sr(2)-O(14) | 2.697(3) |
| Sr(2)-O(8)D | 2.576(3) | Sr(2)-O(7)D | 2.737(3) |
| Angle | (**°**) | Angle | (**°**) |
| O(9)A-Eu(1)-O(5) | 70.62(11) | O(11)-Sr(1)-O(15) | 76.97(11) |
| O(9)A-Eu(1)-O(3) | 89.69(12) | O(11)-Sr(1)-O(12)B | 119.52(11) |
| O(5)-Eu(1)-O(3) | 148.28(12) | O(15)-Sr(1)-O(12)B | 102.38(11) |
| O(9)A-Eu(1)-O(1) | 83.95(12) | O(11)-Sr(1)-O(3) | 106.68(10) |
| O(5)-Eu(1)-O(1) | 76.99(11) | O(15)-Sr(1)-O(3) | 138.02(11) |
| O(3)-Eu(1)-O(1) | 126.77(11) | O(12)B-Sr(1)-O(3) | 110.64(11) |
| O(9)A-Eu(1)-O(7) | 159.04(11) | O(11)-Sr(1)-O(13) | 77.22(11) |
| O(5)-Eu(1)-O(7) | 126.24(10) | O(15)-Sr(1)-O(13) | 145.53(10) |
| O(3)-Eu(1)-O(7) | 79.63(11) | O(12)B-Sr(1)-O(13) | 71.59(10) |
| O(1)-Eu(1)-O(7) | 88.07(11) | O(3)-Sr(1)-O(13) | 71.90(10) |
| O(9)A-Eu(1)-O(10) | 117.18(11) | O(11)-Sr(1)-O(5)A | 102.02(11) |
| O(5)-Eu(1)-O(10) | 89.43(11) | O(15)-Sr(1)-O(5)A | 68.47(10) |
| O(3)-Eu(1)-O(10) | 77.48(10) | O(12)B-Sr(1)-O(5)A | 134.70(10) |
| O(1)-Eu(1)-O(10) | 149.74(10) | O(3)-Sr(1)-O(5)A | 69.94(11) |
| O(7)-Eu(1)-O(10) | 78.28(10) | O(13)-Sr(1)-O(5)A | 139.78(10) |
| O(9)A-Eu(1)-N(1) | 84.48(12) | O(11)-Sr(1)-O(6)A | 142.23(11) |
| O(5)-Eu(1)-N(1) | 135.21(11) | O(15)-Sr(1)-O(6)A | 70.92(10) |
| O(3)-Eu(1)-N(1) | 63.30(11) | O(12)B-Sr(1)-O(6)A | 86.97(10) |
| O(1)-Eu(1)-N(1) | 63.49(11) | O(3)-Sr(1)-O(6)A | 85.40(10) |
| O(7)-Eu(1)-N(1) | 74.62(11) | O(13)-Sr(1)-O(6)A | 139.88(10) |
| O(10)-Eu(1)-N(1) | 135.36(12) | O(5)A-Sr(1)-O(6)A | 47.76(9) |
| O(9)A-Eu(1)-N(2) | 133.70(12) | O(11)-Sr(1)-O(4) | 143.07(10) |
| O(5)-Eu(1)-N(2) | 63.29(12) | O(15)-Sr(1)-O(4) | 139.79(10) |
| O(3)-Eu(1)-N(2) | 133.20(11) | O(12)B-Sr(1)-O(4) | 65.46(10) |
| O(1)-Eu(1)-N(2) | 81.84(11) | O(3)-Sr(1)-O(4) | 47.25(9) |
| O(7)-Eu(1)-N(2) | 63.60(11) | O(13)-Sr(1)-O(4) | 70.00(9) |
| O(10)-Eu(1)-N(2) | 67.90(11) | O(5)A-Sr(1)-O(4) | 92.58(10) |
| N(1)-Eu(1)-N(2) | 126.03(13) | O(6)A-Sr(1)-O(4) | 70.29(10) |
| O(9)A-Eu(1)-O(9) | 67.21(12) | O(11)-Sr(1)-O(11)B | 81.49(10) |
| O(5)-Eu(1)-O(9) | 74.78(11) | O(15)-Sr(1)-O(11)B | 70.66(10) |
| O(3)-Eu(1)-O(9) | 74.69(11) | O(12)B-Sr(1)-O(11)B | 45.00(10) |
| O(1)-Eu(1)-O(9) | 144.85(12) | O(3)-Sr(1)-O(11)B | 150.90(10) |
| O(7)-Eu(1)-O(9) | 125.72(10) | O(13)-Sr(1)-O(11)B | 83.25(9) |
| O(10)-Eu(1)-O(9) | 50.01(10) | O(5)A-Sr(1)-O(11)B | 136.86(10) |
| N(1)-Eu(1)-O(9) | 129.09(11) | O(6)A-Sr(1)-O(11)B | 105.38(9) |
| N(2)-Eu(1)-O(9) | 103.34(11) | O(4)-Sr(1)-O(11)B | 110.31(9) |
| O(2)C-Sr(2)-O(4) | 145.89(12) | O(12)B-Sr(2)-O(13) | 69.33(10) |
| O(2)C-Sr(2)-O(14)D | 138.47(12) | O(2)C-Sr(2)-O(14) | 107.67(11) |
| O(4)-Sr(2)-O(14)D | 75.63(11) | O(4)-Sr(2)-O(14) | 84.72(10) |
| O(2)C-Sr(2)-O(8)D | 89.64(11) | O(14)D-Sr(2)-O(14) | 64.32(12) |
| O(4)-Sr(2)-O(8)D | 96.05(10) | O(8)D-Sr(2)-O(14) | 147.80(11) |
| O(14)D-Sr(2)-O(8)D | 84.60(11) | O(12)B-Sr(2)-O(14) | 117.39(11) |
| O(2)C-Sr(2)-O(12)B | 76.14(11) | O(13)-Sr(2)-O(14) | 48.75(10) |
| O(4)-Sr(2)-O(12)B | 70.02(10) | O(2)C-Sr(2)-O(7)D | 72.53(11) |
| O(14)D-Sr(2)-O(12)B | 145.07(11) | O(4)-Sr(2)-O(7)D | 134.50(10) |
| O(8)D-Sr(2)-O(12)B | 92.69(11) | O(14)D-Sr(2)-O(7)D | 72.57(11) |
| O(2)C-Sr(2)-O(13) | 89.22(11) | O(8)D-Sr(2)-O(7)D | 49.68(9) |
| O(4)-Sr(2)-O(13) | 75.13(10) | O(12)B-Sr(2)-O(7)D | 129.84(10) |
| O(14)D-Sr(2)-O(13) | 108.02(10) | O(13)-Sr(2)-O(7)D | 145.96(9) |
| O(8)D-Sr(2)-O(13) | 161.71(11) | O(14)-Sr(2)-O(7)D | 109.00(10) |
| Complex **4** Bond | Dist. | Bond | Dist. |
| Tb(1)-O(9)A | 236.4(3) | Sr(1)-O(11) | 251.6(3) |
| Tb(1)-O(5) | 237.0(3) | Sr(1)-O(15) | 254.7(3) |
| Tb(1)-O(1) | 238.1(3) | Sr(1)-O(12)B | 257.8(3) |
| Tb(1)-O(3) | 238.1(3) | Sr(1)-O(3) | 260.3(3) |
| Tb(1)-O(7) | 241.7(3) | Sr(1)-O(13) | 261.4(3) |
| Tb(1)-O(10) | 246.2(3) | Sr(1)-O(5)A | 261.7(3) |
| Tb(1)-N(2) | 250.8(3) | Sr(1)-O(6)A | 285.6(3) |
| Tb(1)-N(1) | 251.0(3) | Sr(1)-O(4) | 288.4(3) |
| Tb(1)-O(9) | 274.9(3) | Sr(1)-O(11)B | 310.5(3) |
| Sr(2)-O(2)C | 246.6(3) | Sr(2)-O(12)B | 264.7(3) |
| Sr(2)-O(4) | 253.9(3) | Sr(2)-O(13) | 268.0(3) |
| Sr(2)-O(8)D | 257.1(3) | Sr(2)-O(14) | 270.0(3) |
| Sr(2)-O(14)D | 257.6(3) | Sr(2)-O(7)D | 275.5(3) |
| Angle | (**°**) | Angle | (**°**) |
| O(9)A-Tb(1)-O(5) | 70.86(10) | O(11)-Sr(1)-O(15) | 77.36(10) |
| O(9)A-Tb(1)-O(1) | 84.20(11) | O(11)-Sr(1)-O(12)B | 119.04(10) |
| O(5)-Tb(1)-O(1) | 77.38(10) | O(15)-Sr(1)-O(12)B | 101.09(10) |
| O(9)A-Tb(1)-O(3) | 88.86(11) | O(11)-Sr(1)-O(3) | 106.86(10) |
| O(5)-Tb(1)-O(3) | 146.74(10) | O(15)-Sr(1)-O(3) | 138.21(10) |
| O(1)-Tb(1)-O(3) | 127.84(10) | O(12)B-Sr(1)-O(3) | 111.47(9) |
| O(9)A-Tb(1)-O(7) | 158.36(10) | O(11)-Sr(1)-O(13) | 77.14(10) |
| O(5)-Tb(1)-O(7) | 127.01(10) | O(15)-Sr(1)-O(13) | 145.48(10) |
| O(1)-Tb(1)-O(7) | 88.17(10) | O(12)B-Sr(1)-O(13) | 72.02(10) |
| O(3)-Tb(1)-O(7) | 79.90(10) | O(3)-Sr(1)-O(13) | 72.12(9) |
| O(9)A-Tb(1)-O(10) | 115.91(10) | O(11)-Sr(1)-O(5)A | 102.70(10) |
| O(5)-Tb(1)-O(10) | 88.40(10) | O(15)-Sr(1)-O(5)A | 68.74(10) |
| O(1)-Tb(1)-O(10) | 150.21(10) | O(12)B-Sr(1)-O(5)A | 134.10(10) |
| O(3)-Tb(1)-O(10) | 76.84(10) | O(3)-Sr(1)-O(5)A | 69.84(9) |
| O(7)-Tb(1)-O(10) | 79.65(10) | O(13)-Sr(1)-O(5)A | 140.09(9) |
| O(9)A-Tb(1)-N(2) | 134.23(11) | O(11)-Sr(1)-O(6)A | 142.60(10) |
| O(5)-Tb(1)-N(2) | 63.60(10) | O(15)-Sr(1)-O(6)A | 70.92(10) |
| O(1)-Tb(1)-N(2) | 81.83(11) | O(12)B-Sr(1)-O(6)A | 86.69(9) |
| O(3)-Tb(1)-N(2) | 133.10(11) | O(3)-Sr(1)-O(6)A | 85.18(9) |
| O(7)-Tb(1)-N(2) | 63.99(10) | O(13)-Sr(1)-O(6)A | 139.63(9) |
| O(10)-Tb(1)-N(2) | 68.39(11) | O(5)A-Sr(1)-O(6)A | 47.42(9) |
| O(9)A-Tb(1)-N(1) | 83.83(11) | O(11)-Sr(1)-O(4) | 143.01(9) |
| O(5)-Tb(1)-N(1) | 135.74(10) | O(15)-Sr(1)-O(4) | 139.52(10) |
| O(1)-Tb(1)-N(1) | 64.12(10) | O(12)B-Sr(1)-O(4) | 66.01(9) |
| O(3)-Tb(1)-N(1) | 63.75(10) | O(3)-Sr(1)-O(4) | 47.38(8) |
| O(7)-Tb(1)-N(1) | 74.63(10) | O(13)-Sr(1)-O(4) | 69.88(9) |
| O(10)-Tb(1)-N(1) | 135.78(11) | O(5)A-Sr(1)-O(4) | 92.44(9) |
| N(2)-Tb(1)-N(1) | 126.68(11) | O(6)A-Sr(1)-O(4) | 70.18(9) |
| O(9)A-Tb(1)-O(9) | 67.06(12) | O(11)-Sr(1)-O(11)B | 81.32(10) |
| O(5)-Tb(1)-O(9) | 74.39(10) | O(15)-Sr(1)-O(11)B | 69.72(9) |
| O(1)-Tb(1)-O(9) | 144.84(10) | O(12)B-Sr(1)-O(11)B | 44.64(9) |
| O(3)-Tb(1)-O(9) | 73.46(9) | O(3)-Sr(1)-O(11)B | 151.57(9) |
| O(7)-Tb(1)-O(9) | 125.74(9) | O(13)-Sr(1)-O(11)B | 83.74(9) |
| O(10)-Tb(1)-O(9) | 48.90(9) | O(5)A-Sr(1)-O(11)B | 136.10(9) |
| N(2)-Tb(1)-O(9) | 103.48(10) | O(6)A-Sr(1)-O(11)B | 104.99(9) |
| N(1)-Tb(1)-O(9) | 128.25(10) | O(4)-Sr(1)-O(11)B | 110.50(8) |
| O(2)C-Sr(2)-O(4) | 145.43(10) | O(2)C-Sr(2)-O(14) | 107.02(11) |
| O(2)C-Sr(2)-O(8)D | 90.33(11) | O(4)-Sr(2)-O(14) | 83.81(10) |
| O(4)-Sr(2)-O(8)D | 97.42(10) | O(8)D-Sr(2)-O(14) | 147.54(10) |
| O(2)C-Sr(2)-O(14)D | 138.37(11) | O(14)D-Sr(2)-O(14) | 64.35(12) |
| O(4)-Sr(2)-O(14)D | 76.10(10) | O(12)B-Sr(2)-O(14) | 117.67(9) |
| O(8)D-Sr(2)-O(14)D | 84.35(10) | O(13)-Sr(2)-O(14) | 48.44(9) |
| O(2)C-Sr(2)-O(12)B | 75.74(10) | O(2)C-Sr(2)-O(7)D | 73.24(10) |
| O(4)-Sr(2)-O(12)B | 70.28(9) | O(4)-Sr(2)-O(7)D | 135.29(9) |
| O(8)D-Sr(2)-O(12)B | 92.80(10) | O(8)D-Sr(2)-O(7)D | 49.30(9) |
| O(14)D-Sr(2)-O(12)B | 145.61(10) | O(14)D-Sr(2)-O(7)D | 72.17(9) |
| O(2)C-Sr(2)-O(13) | 88.33(10) | O(12)B-Sr(2)-O(7)D | 129.58(9) |
| O(4)-Sr(2)-O(13) | 74.38(9) | O(13)-Sr(2)-O(7)D | 145.59(9) |
| O(8)D-Sr(2)-O(13) | 162.47(9) | O(14)-Sr(2)-O(7)D | 108.95(9) |
| O(14)D-Sr(2)-O(13) | 108.00(9) | O(12)B-Sr(2)-O(13) | 69.93(9) |
| Complex **5:** Bond | Dist. | Bond | Dist. |
| Eu(1)-O(5) | 2.3967(19) | Ba(2)-O(2)C | 2.609(2) |
| Eu(1)-O(1) | 2.3974(19) | Ba(2)-O(14)D | 2.704(2) |
| Eu(1)-O(9)A | 2.399(2) | Ba(2)-O(4) | 2.727(2) |
| Eu(1)-O(3) | 2.4043(19) | Ba(2)-O(8)D | 2.732(2) |
| Eu(1)-O(7) | 2.4381(18) | Ba(2)-O(12)B | 2.7562(19) |
| Eu(1)-O(10) | 2.4920(19) | Ba(2)-O(14) | 2.825(2) |
| Eu(1)-N(2) | 2.538(2) | Ba(2)-O(13) | 2.866(2) |
| Eu(1)-N(1) | 2.546(2) | Ba(2)-O(7)D | 2.9006(19) |
| Eu(1)-O(9) | 2.808(2) | Ba(1)-O(12)B | 2.776(2) |
| Ba(1)-O(11) | 2.6662(19) | Ba(1)-O(13) | 2.791(2) |
| Ba(1)-O(15) | 2.725(2) | Ba(1)-O(6)A | 2.928(2) |
| Ba(1)-O(3) | 2.7427(18) | Ba(1)-O(4) | 2.989(2) |
| Ba(1)-O(5)A | 2.7726(19) | Ba(1)-O(11)B | 3.133(2) |
| Angle | (**°**) | Angle | (**°**) |
| O(5)-Eu(1)-O(1) | 79.43(7) | O(2)C-Ba(2)-O(14)D | 139.56(7) |
| O(5)-Eu(1)-O(9)A | 70.15(7) | O(2)C-Ba(2)-O(4) | 143.96(6) |
| O(1)-Eu(1)-O(9)A | 82.77(7) | O(14)D-Ba(2)-O(4) | 75.95(7) |
| O(5)-Eu(1)-O(3) | 147.74(6) | O(2)C-Ba(2)-O(8)D | 90.71(7) |
| O(1)-Eu(1)-O(3) | 125.96(6) | O(14)D-Ba(2)-O(8)D | 83.46(7) |
| O(9)A-Eu(1)-O(3) | 91.80(7) | O(4)-Ba(2)-O(8)D | 101.96(6) |
| O(5)-Eu(1)-O(7) | 125.93(6) | O(2)C-Ba(2)-O(12)B | 75.62(7) |
| O(1)-Eu(1)-O(7) | 86.27(7) | O(14)D-Ba(2)-O(12)B | 144.38(7) |
| O(9)A-Eu(1)-O(7) | 158.37(7) | O(4)-Ba(2)-O(12)B | 70.30(6) |
| O(3)-Eu(1)-O(7) | 79.65(7) | O(8)D-Ba(2)-O(12)B | 92.52(6) |
| O(5)-Eu(1)-O(10) | 86.59(7) | O(2)C-Ba(2)-O(14) | 106.41(7) |
| O(1)-Eu(1)-O(10) | 151.59(7) | O(14)D-Ba(2)-O(14) | 67.15(7) |
| O(9)A-Eu(1)-O(10) | 115.58(7) | O(4)-Ba(2)-O(14) | 78.97(6) |
| O(3)-Eu(1)-O(10) | 77.24(7) | O(8)D-Ba(2)-O(14) | 149.62(6) |
| O(7)-Eu(1)-O(10) | 82.11(6) | O(12)B-Ba(2)-O(14) | 115.69(6) |
| O(5)-Eu(1)-N(2) | 63.07(7) | O(2)C-Ba(2)-O(13) | 86.76(7) |
| O(1)-Eu(1)-N(2) | 81.35(7) | O(14)D-Ba(2)-O(13) | 108.74(6) |
| O(9)A-Eu(1)-N(2) | 132.52(7) | O(4)-Ba(2)-O(13) | 71.65(6) |
| O(3)-Eu(1)-N(2) | 133.02(7) | O(8)D-Ba(2)-O(13) | 163.62(6) |
| O(7)-Eu(1)-N(2) | 63.33(7) | O(12)B-Ba(2)-O(13) | 71.18(6) |
| O(10)-Eu(1)-N(2) | 70.26(7) | O(14)-Ba(2)-O(13) | 45.58(6) |
| O(5)-Eu(1)-N(1) | 137.35(7) | O(2)C-Ba(2)-O(7)D | 75.99(6) |
| O(1)-Eu(1)-N(1) | 63.31(7) | O(14)D-Ba(2)-O(7)D | 71.12(6) |
| O(9)A-Eu(1)-N(1) | 84.60(7) | O(4)-Ba(2)-O(7)D | 135.91(6) |
| O(3)-Eu(1)-N(1) | 62.65(7) | O(8)D-Ba(2)-O(7)D | 46.50(6) |
| O(7)-Eu(1)-N(1) | 73.82(7) | O(12)B-Ba(2)-O(7)D | 128.82(6) |
| O(10)-Eu(1)-N(1) | 135.93(7) | O(14)-Ba(2)-O(7)D | 112.76(6) |
| N(2)-Eu(1)-N(1) | 125.59(7) | O(13)-Ba(2)-O(7)D | 147.01(6) |
| O(5)-Eu(1)-O(9) | 73.85(6) | O(5)A-Ba(1)-O(6)A | 45.72(5) |
| O(1)-Eu(1)-O(9) | 145.26(7) | O(12)B-Ba(1)-O(6)A | 88.58(6) |
| O(9)A-Eu(1)-O(9) | 67.61(8) | O(13)-Ba(1)-O(6)A | 140.23(6) |
| O(3)-Eu(1)-O(9) | 74.57(6) | O(11)-Ba(1)-O(4) | 140.38(6) |
| O(7)-Eu(1)-O(9) | 127.51(6) | O(15)-Ba(1)-O(4) | 141.34(6) |
| O(10)-Eu(1)-O(9) | 48.14(6) | O(3)-Ba(1)-O(4) | 45.37(5) |
| N(2)-Eu(1)-O(9) | 105.10(6) | O(5)A-Ba(1)-O(4) | 90.30(6) |
| N(1)-Eu(1)-O(9) | 127.79(6) | O(12)B-Ba(1)-O(4) | 66.28(5) |
| O(11)-Ba(1)-O(15) | 77.36(7) | O(13)-Ba(1)-O(4) | 68.93(6) |
| O(11)-Ba(1)-O(3) | 104.84(6) | O(6)A-Ba(1)-O(4) | 71.52(6) |
| O(15)-Ba(1)-O(3) | 136.14(6) | O(11)-Ba(1)-O(11)B | 84.96(6) |
| O(11)-Ba(1)-O(5)A | 102.32(6) | O(15)-Ba(1)-O(11)B | 74.79(6) |
| O(15)-Ba(1)-O(5)A | 68.19(6) | O(3)-Ba(1)-O(11)B | 148.60(6) |
| O(3)-Ba(1)-O(5)A | 68.63(6) | O(5)A-Ba(1)-O(11)B | 139.35(6) |
| O(11)-Ba(1)-O(12)B | 121.16(6) | O(12)B-Ba(1)-O(11)B | 43.41(5) |
| O(15)-Ba(1)-O(12)B | 105.49(6) | O(13)-Ba(1)-O(11)B | 83.30(6) |
| O(3)-Ba(1)-O(12)B | 109.68(6) | O(6)A-Ba(1)-O(11)B | 106.32(6) |
| O(5)A-Ba(1)-O(12)B | 134.07(6) | O(4)-Ba(1)-O(11)B | 109.47(5) |
| O(11)-Ba(1)-O(13) | 76.81(6) | O(5)A-Ba(1)-O(13) | 137.36(6) |
| O(15)-Ba(1)-O(13) | 147.31(6) | O(12)B-Ba(1)-O(13) | 72.01(6) |
| O(3)-Ba(1)-O(13) | 70.51(6) | O(11)-Ba(1)-O(6)A | 141.26(6) |
| O(3)-Ba(1)-O(6)A | 84.81(6) | O(15)-Ba(1)-O(6)A | 70.55(6) |
| Complex **6:** Bond | Dist. | Bond | Dist. |
| Tb(1)-O(9)A | 2.350(2) | Ba(1)-O(11) | 2.661(2) |
| Tb(1)-O(5) | 2.3681(19) | Ba(1)-O(15) | 2.723(2) |
| Tb(1)-O(1) | 2.374(2) | Ba(1)-O(3) | 2.7526(19) |
| Tb(1)-O(3) | 2.3832(19) | Ba(1)-O(5)A | 2.7677(19) |
| Tb(1)-O(7) | 2.4152(18) | Ba(1)-O(12)B | 2.789(2) |
| Tb(1)-O(10) | 2.439(2) | Ba(1)-O(13) | 2.792(2) |
| Tb(1)-N(2) | 2.508(2) | Ba(1)-O(6)A | 2.939(2) |
| Tb(1)-N(1) | 2.513(2) | Ba(1)-O(4) | 2.966(2) |
| Ba(2)-O(2)C | 2.601(2) | Ba(1)-O(11)B | 3.138(2) |
| Ba(2)-O(14)D | 2.701(2) | Ba(2)-O(12)B | 2.754(2) |
| Ba(2)-O(8)D | 2.727(2) | Ba(2)-O(14) | 2.817(2) |
| Ba(2)-O(4) | 2.728(2) | Ba(2)-O(13) | 2.872(2) |
| Ba(2)-O(7)D | 2.9117(18) |  |  |
| Angle | (**°**) | Angle | (**°**) |
| O(9)A-Tb(1)-O(5) | 70.28(7) | O(11)-Ba(1)-O(15) | 77.54(7) |
| O(9)A-Tb(1)-O(1) | 83.00(8) | O(11)-Ba(1)-O(3) | 105.10(6) |
| O(5)-Tb(1)-O(1) | 80.25(7) | O(15)-Ba(1)-O(3) | 136.67(6) |
| O(9)A-Tb(1)-O(3) | 90.91(8) | O(11)-Ba(1)-O(5)A | 102.83(6) |
| O(5)-Tb(1)-O(3) | 145.43(6) | O(15)-Ba(1)-O(5)A | 68.16(6) |
| O(1)-Tb(1)-O(3) | 127.34(7) | O(3)-Ba(1)-O(5)A | 69.17(6) |
| O(9)A-Tb(1)-O(7) | 157.42(7) | O(11)-Ba(1)-O(12)B | 120.74(6) |
| O(5)-Tb(1)-O(7) | 127.20(6) | O(15)-Ba(1)-O(12)B | 104.39(6) |
| O(1)-Tb(1)-O(7) | 86.23(7) | O(3)-Ba(1)-O(12)B | 110.14(6) |
| O(3)-Tb(1)-O(7) | 79.96(7) | O(5)A-Ba(1)-O(12)B | 133.57(6) |
| O(9)A-Tb(1)-O(10) | 113.64(8) | O(11)-Ba(1)-O(13) | 76.99(6) |
| O(5)-Tb(1)-O(10) | 84.31(7) | O(15)-Ba(1)-O(13) | 147.34(6) |
| O(1)-Tb(1)-O(10) | 151.84(7) | O(3)-Ba(1)-O(13) | 70.39(6) |
| O(3)-Tb(1)-O(10) | 76.95(7) | O(5)A-Ba(1)-O(13) | 137.93(6) |
| O(7)-Tb(1)-O(10) | 84.56(7) | O(12)B-Ba(1)-O(13 | 72.25(6) |
| O(9)A-Tb(1)-N(2) | 132.99(8) | O(11)-Ba(1)-O(6)A | 141.56(6) |
| O(5)-Tb(1)-N(2) | 63.58(7) | O(15)-Ba(1)-O(6)A | 70.49(6) |
| O(1)-Tb(1)-N(2) | 80.99(7) | O(3)-Ba(1)-O(6)A | 85.07(6) |
| O(3)-Tb(1)-N(2) | 133.13(7) | O(5)A-Ba(1)-O(6)A | 45.57(5) |
| O(7)-Tb(1)-N(2) | 63.97(7) | O(12)B-Ba(1)-O(6)A | 88.14(6) |
| O(10)-Tb(1)-N(2) | 71.05(7) | O(13)-Ba(1)-O(6)A | 139.95(6) |
| O(9)A-Tb(1)-N(1) | 83.41(7) | O(11)-Ba(1)-O(4) | 140.73(6) |
| O(5)-Tb(1)-N(1) | 137.93(7) | O(15)-Ba(1)-O(4) | 140.99(6) |
| O(1)-Tb(1)-N(1) | 64.02(7) | O(3)-Ba(1)-O(4) | 45.56(5) |
| O(3)-Tb(1)-N(1) | 63.31(7) | O(5)A-Ba(1)-O(4) | 90.43(6) |
| O(7)-Tb(1)-N(1) | 74.04(7) | O(12)B-Ba(1)-O(4) | 66.56(6) |
| O(10)-Tb(1)-N(1) | 137.24(7) | O(13)-Ba(1)-O(4) | 68.87(6) |
| N(2)-Tb(1)-N(1) | 126.47(7) | O(6)A-Ba(1)-O(4) | 71.33(6) |
| O(2)C-Ba(2)-O(14)D | 139.14(7) | O(11)-Ba(1)-O(11)B | 84.90(6) |
| O(2)C-Ba(2)-O(8)D | 91.40(8) | O(15)-Ba(1)-O(11)B | 73.45(6) |
| O(14)D-Ba(2)-O(8)D | 83.26(7) | O(3)-Ba(1)-O(11)B | 149.29(5) |
| O(2)C-Ba(2)-O(4) | 143.42(7) | O(5)A-Ba(1)-O(11)B | 137.84(6) |
| O(14)D-Ba(2)-O(4) | 76.65(6) | O(12)B-Ba(1)-O(11)B | 43.25(5) |
| O(8)D-Ba(2)-O(4) | 102.74(6) | O(13)-Ba(1)-O(11)B | 84.23(6) |
| O(2)C-Ba(2)-O(12)B | 75.46(7) | O(6)A-Ba(1)-O(11)B | 105.25(6) |
| O(14)D-Ba(2)-O(12)B | 144.99(7) | O(4)-Ba(1)-O(11)B | 109.66(5) |
| O(8)D-Ba(2)-O(12)B | 92.48(6) | O(8)D-Ba(2)-O(13) | 163.96(6) |
| O(4)-Ba(2)-O(12)B | 70.44(6) | O(4)-Ba(2)-O(13) | 71.13(6) |
| O(2)C-Ba(2)-O(14) | 105.17(7) | O(12)B-Ba(2)-O(13) | 71.54(6) |
| O(14)D-Ba(2)-O(14) | 67.29(8) | O(14)-Ba(2)-O(13) | 45.69(6) |
| O(8)D-Ba(2)-O(14) | 149.38(7) | O(2)C-Ba(2)-O(7)D | 76.57(7) |
| O(4)-Ba(2)-O(14) | 79.20(6) | O(14)D-Ba(2)-O(7)D | 70.57(6) |
| O(12)B-Ba(2)-O(14) | 116.32(6) | O(8)D-Ba(2)-O(7)D | 46.38(5) |
| O(2)C-Ba(2)-O(13) | 86.02(7) | O(4)-Ba(2)-O(7)D | 136.42(6) |
| O(14)D-Ba(2)-O(13) | 10 8.88(6) | O(12)B-Ba(2)-O(7)D | 128.67(6) |
| O(13)-Ba(2)-O(7)D | 146.80(6) | O(14)-Ba(2)-O(7)D | 112.10(6) |
| Symmetry codes for complex **1**: A: -x+1, -y+1, -z+1; B: -x+1, -y+2, -z+1; C: x-1, y, z; D: x, y+1, z; E: x+1, y, z; F: x, y-1, z  complex **2**: A: -x+1, -y+2, -z+1; B: x+1, y, z; C: x, y-1, z; D: x-1, y, z; E: x, y+1, z  complex **3**: A: -x+1, -y, -z+2; B: -x, -y, -z+2; C: x-1, y, z; D: -x, -y, -z+1; E: x+1, y, z  complex **4**: A: -x+1, -y, -z+2; B: -x, -y, -z+2; C: x-1, y, z; D: -x, -y, -z+1; E: x+1, y, z  complex **5**: A: -x+1, -y, -z+2; B: -x, -y, -z+2; C: x-1, y, z; D: -x, -y, -z+1; E: x+1, y, z  complex **6**: A: -x+1, -y, -z+2; B: -x, -y, -z+2; C: x-1, y, z; D: -x, -y, -z+1; E: x+1, y, z | | | |

**Table S2**. Intermolecular Hydrogen Bond Lengths (Å) and Bond Angles (°) of Complexes **1**-**6**

| Complex **1:** D–H···A | d(D–H) (Å) | d(H···A) (Å) | d(D···A) (Å) | ∠DHA(°) |
| --- | --- | --- | --- | --- |
| C(4)-H(4)...O(14)G | 0.95 | 2.49 | 3.163(6) | 127.6 |
| O(12)-H(12A)...O(5) | 0.83 | 2.21 | 2.617(3) | 110.7 |
| O(13)-H(13A)...O(10) | 0.85 | 2.54 | 3.317(4) | 151.5 |
| O(13)-H(13B)...O(3)C | 0.85 | 2.12 | 2.726(3) | 127.5 |
| O(14)-H(14)...O(8) | 0.84 | 2.13 | 2.863(6) | 145.7 |
| C(27)-H(27A)...O(4)H | 0.98 | 2.34 | 3.321(9) | 175.6 |
| C(27)-H(27B)...O(14)K | 0.98 | 2.05 | 2.982(13) | 159.1 |
| C(27)-H(27C)...O(2)F | 0.98 | 2.13 | 2.909(9) | 135.5 |
| Complex **2**: D–H···A | d(D–H) (Å) | d(H···A) (Å) | d(D···A) (Å) | ∠DHA(°) |
| C(4)-H(4)...O(14)F | 0.93 | 2.52 | 3.175(6) | 128.1 |
| O(12)-H(12)...O(7)C | 0.82 | 1.88 | 2.635(3) | 151.9 |
| O(12)-H(12)...O(8)C | 0.82 | 2.64 | 3.387(3) | 151.6 |
| O(14)-H(14)...O(6)G | 0.82 | 2.14 | 2.867(6) | 147.9 |
| C(27)-H(27A)...O(14)H | 0.96 | 2.08 | 2.983(11) | 156.6 |
| C(27)-H(27B)...O(4)K | 0.96 | 2.36 | 3.308(8) | 171.8 |
| C(27)-H(27C)...O(2)G | 0.96 | 2.12 | 2.914(8) | 138.9 |
| O(13)-H(13B)...O(3)D | 0.85 | 1.92 | 2.733(3) | 159.2 |
| Complex **3**: D–H···A | d(D–H) (Å) | d(H···A) (Å) | d(D···A) (Å) | ∠DHA(°) |
| C(9)-H(9)...O(8)F | 0.93 | 2.37 | 3.194(6) | 147.3 |
| C(11)-H(11)...O(6)G | 0.93 | 2.33 | 3.095(5) | 139.5 |
| O(15)-H(15B)...O(13)B | 0.85 | 2.03 | 2.760(4) | 143.1 |
| C(27)-H(27)...O(10) | 1.01(5) | 2.60(5) | 3.344(6) | 130(3) |
| Complex **4**: D–H···A | d(D–H) (Å) | d(H···A) (Å) | d(D···A) (Å) | ∠DHA(°) |
| C(9)-H(9)...O(8)F | 93 | 243 | 324.4(6) | 146.8 |
| C(11)-H(11)...O(6)G | 93 | 236 | 312.1(5) | 138.8 |
| O(15)-H(15B)...O(13)B | 85 | 204 | 277.4(4) | 143.8 |
| Complex **5**: D–H···A | d(D–H) (Å) | d(H···A) (Å) | d(D···A) (Å) | ∠DHA(°) |
| C(9)-H(9)...O(8)F | 0.93 | 2.36 | 3.226(4) | 153.9 |
| C(11)-H(11)...O(6)G | 0.93 | 2.30 | 3.128(4) | 147.5 |
| O(15)-H(15B)...O(13)B | 0.87 | 2.03 | 2.777(3) | 143.2 |
| O(15)-H(15A)...O(1)A | 0.88 | 1.96 | 2.793(3) | 158.2 |
| Complex 6: D–H···A | d(D–H) (Å) | d(H···A) (Å) | d(D···A) (Å) | ∠DHA(°) |
| C(9)-H(9)...O(8)F | 0.93 | 2.37 | 3.227(4) | 153.6 |
| C(11)-H(11)...O(6)G | 0.93 | 2.31 | 3.128(4) | 147.0 |
| O(15)-H(15B)...O(13)B | 0.87 | 2.03 | 2.778(3) | 144.2 |
| O(15)-H(15A)...O(1)A | 0.87 | 1.95 | 2.787(3) | 160.9 |
| Symmetry codes for complex **1**: A: -x+1, -y+1, -z+1; B: -x+1, -y+2, -z+1; C: x-1, y, z; D: x, y+1, z; E: x+1, y, z; F: x, y-1, z; G: -x+1, -y, -z+2; H: x-1, y-1, z; K: -x, -y-1, -z+2  complex **2**: A: -x+1, -y+2, -z+1; B: x+1, y, z; C: x, y-1, z; D: x-1, y, z; E: x, y+1, z; F: x, y, z+1; G: -x+1, -y+1, -z+1; H: -x+2, -y+1, -z; K: -x+2, -y+1, -z+1  complex **3**: A: -x+1, -y, -z+2; B: -x, -y, -z+2; C: x-1, y, z; D: -x, -y, -z+;1 E: x+1, y, z; F: x+1/2, -y+1/2, z+1/2; G: x-1/2, -y+1/2, z-1/2  complex **4**: A: -x+1, -y, -z+2; B: -x, -y, -z+2; C: x-1, y, z; D: -x, -y, -z+1; E: x+1, y, z; F: x+1/2, -y+1/2, z+1/2; G: x-1/2, -y+1/2, z-1/2  complex **5**: A: -x+1, -y, -z+2; B: -x, -y, -z+2; C: x-1, y, z; D: -x, -y, -z+1; E: x+1, y, z; F: x+1/2, -y+1/2, z+1/2; G: x-1/2, -y+1/2, z-1/2  complex **6**: A: -x+1, -y, -z+2; B: -x, -y, -z+2; C: x-1, y, z; D: -x, -y, -z+1; E: x+1, y, z; F: x+1/2, -y+1/2, z+1/2; G: x-1/2, -y+1/2, z-1/2 | | | | |

**Table S3.** Fluorescence lifetime and quantum yield of complexes **1**-**6**

|  | **1** | **2** | **3** | **4** | **5** | **6** |
| --- | --- | --- | --- | --- | --- | --- |
| Quantum yield | 63.01% | 4.61% | 60.61% | 0.65% | 87.39% | 5.56% |
| Fluorescence lifetime(μs) | 1930.94 | 145.859 | 2049.48 | 109.24 | 2413.04 | 208.95 |
| Attenuation type | Single exponential decay | Double exponential decay | Single exponential decay | Double exponential decay | Single exponential decay | Double exponential decay |

**Table S4.** The HOMO and LUMO orbital energies of monomers and dimers of **1** and **5**.

| Cluster | Complex | HOMO/eV | LUMO/eV | gap/eV |
| --- | --- | --- | --- | --- |
| monomer | **1** | -2.28 | -1.89 | 0.39 |
|  | **5** | -2.15 | -1.76 | 0.39 |
| dimer | **1** | -1.75 | -1.53 | 0.22 |
|  | **5** | -1.61 | -1.42 | 0.19 |

Calculation details: The geometries of monomers and dimers of complexes **1** and **5** were extracted from the crystal structures. All atoms were kept fixed and only hydrogen atoms are optimized. All geometries were optimized under PBE0/def2-SVP level were performed with the Gaussian 16 (C.01) program.


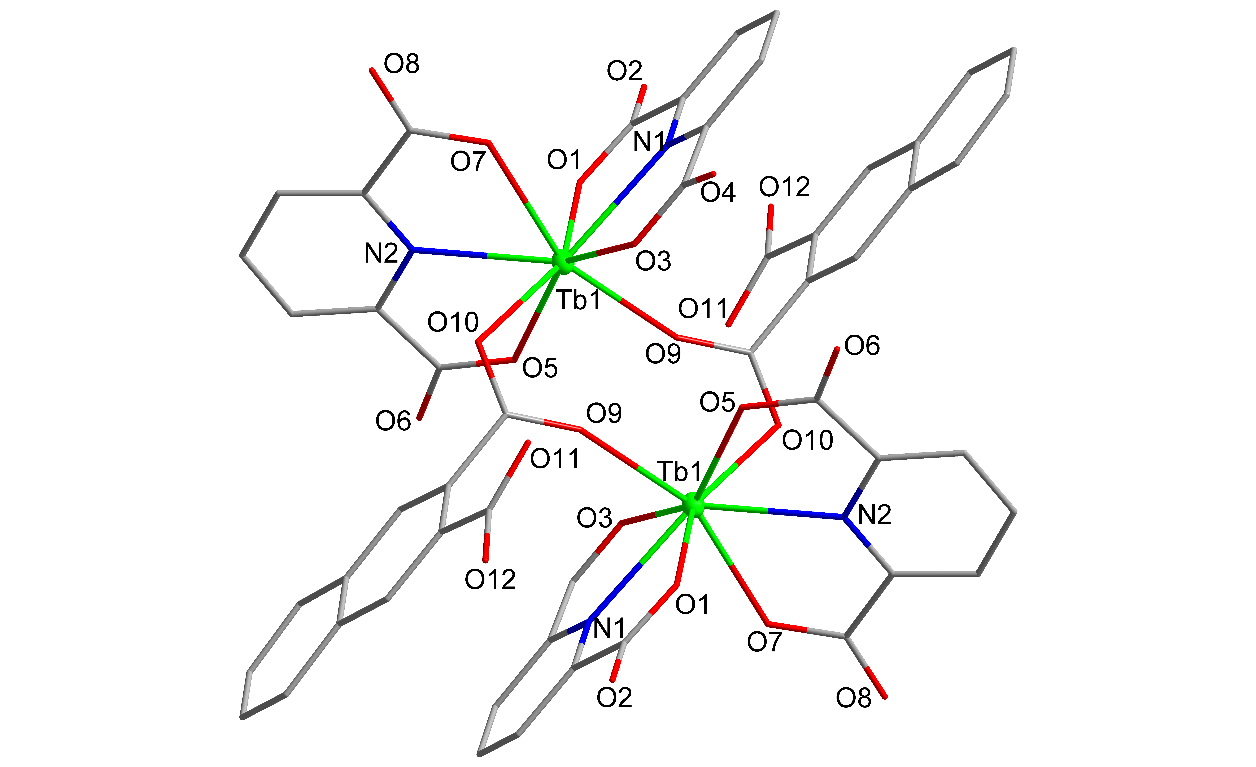


**Figure S1.** The coordination environment of Tb1 in complex **6**.

**Figure S2.** The PXRD spectrum of complexes **1**-**6**.


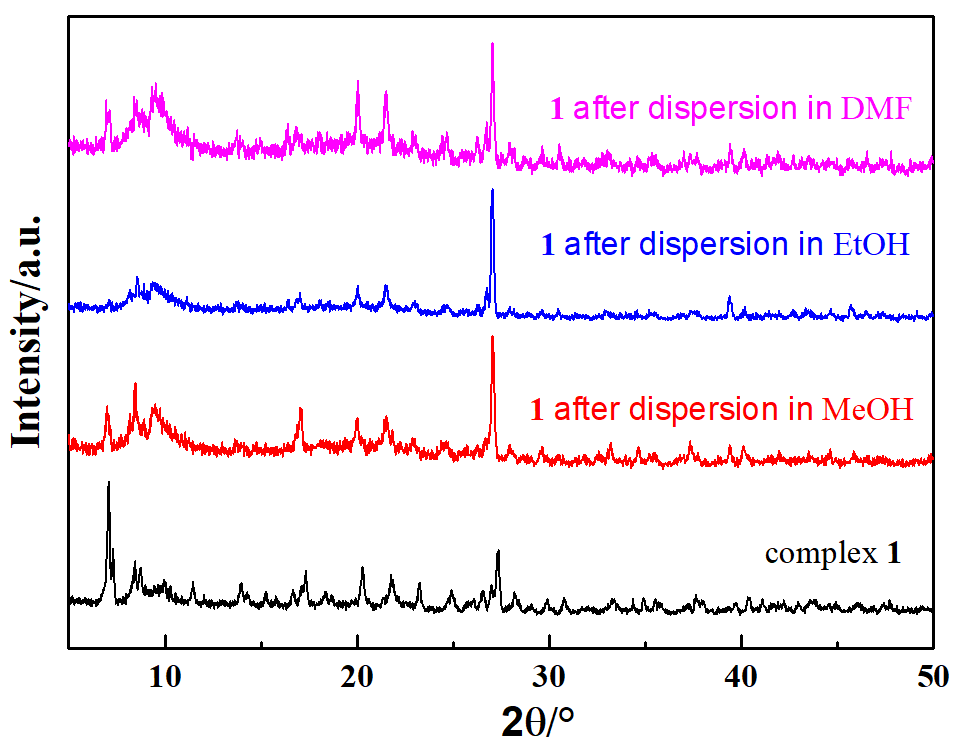

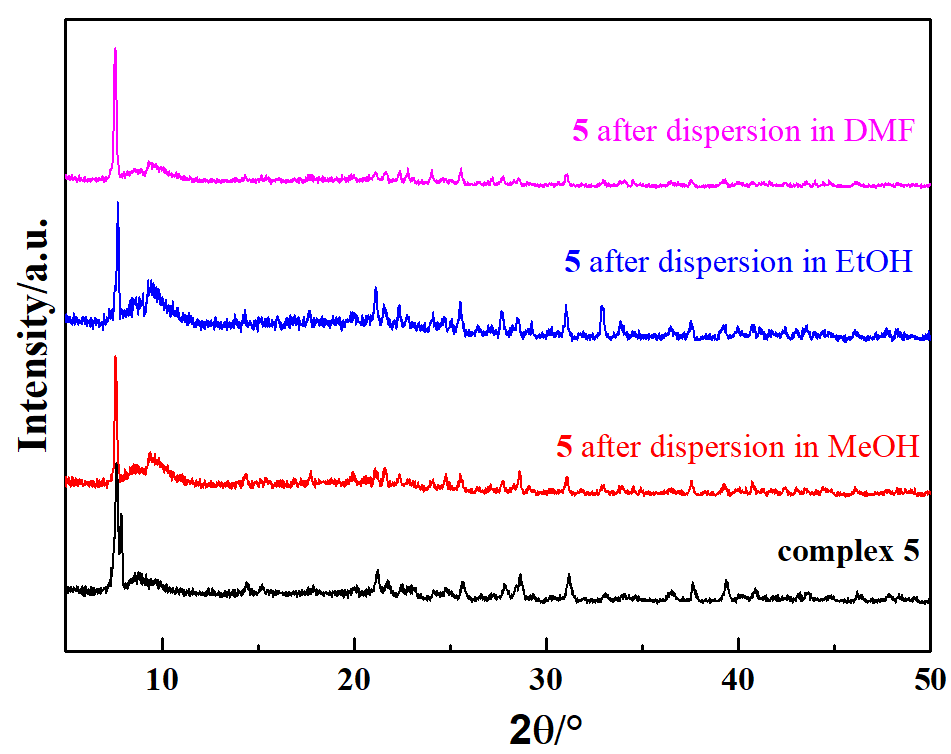


**Figure S3**. The PXRD spectrum of complexes **1** and **5** before and after dispersion in ethanol, methanol, and DMF.


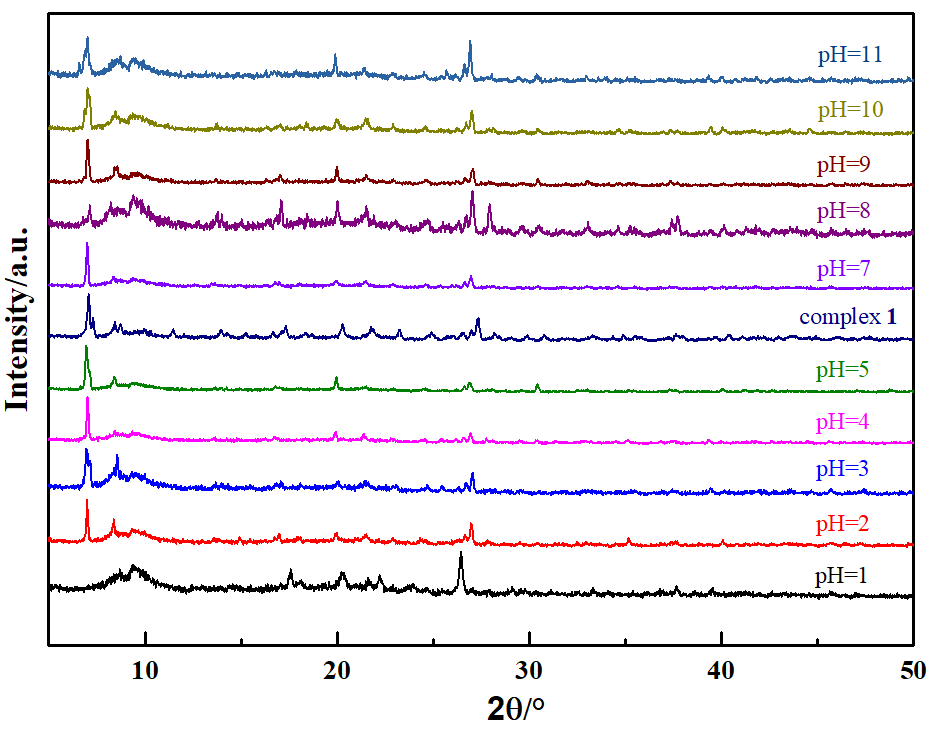

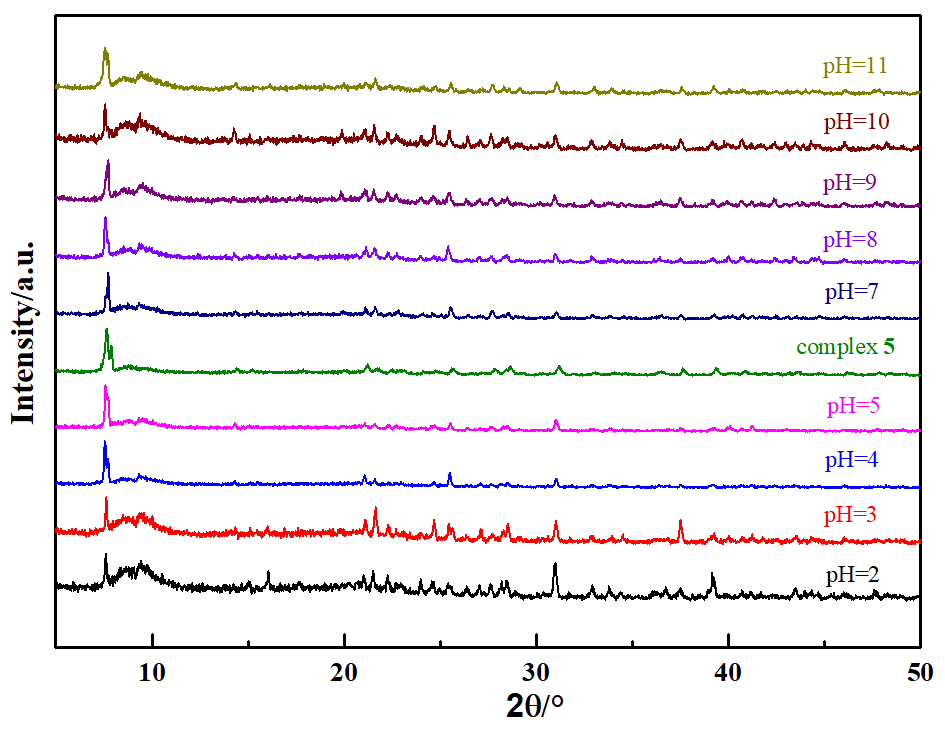


**Figure S4**. The PXRD spectrum of complexes **1** and **5** before and after dispersion in aqueous solutions with different pH values.

**Figure S5.** TGA diagrams of complexes **1**-**6**.

**Figure S6.** IR diagrams of complexes **1** and **2**.

**Figure S7.** IR diagrams of complexes **3** and **4**.

**Figure S8.** IR diagrams of complexes **5** and **6**.

(a)(b)

(c)(d)

(e)(f)

**Figure S9.** Solid state PL spectra of complexes **1**-**6**.


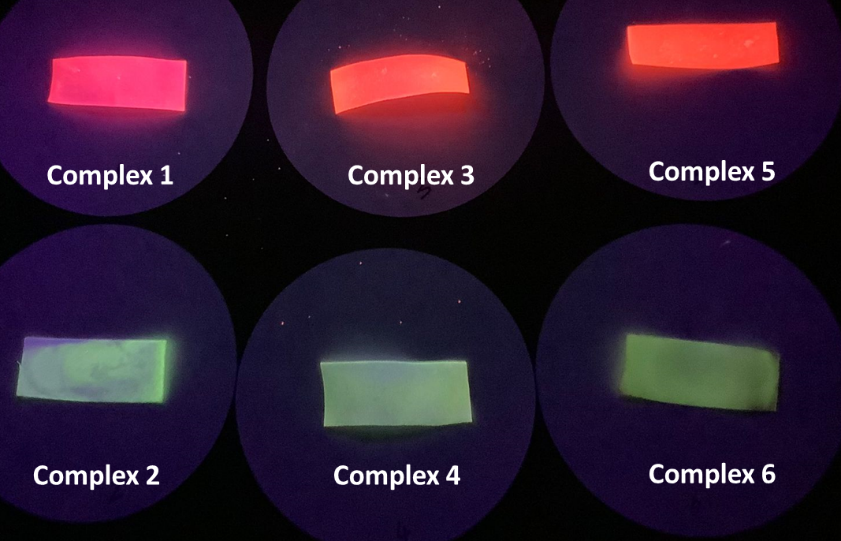


**Figure S10**. Photograph of test paper samples under 254 nm UV light.

**Figure S11.** Solid state fluorescence lifetime of complexes **1**-**6**.

(a)

(b)

**Figure S12.** (a) and (b) are 2D plane of luminescent intensities of **1** and **5** in aqueous solution with various metal ions, respectively.

(a)

(b)

(c)

**Figure S13.** (a) 2D plane of luminescent intensities of **2** in aqueous solution with various metal ions. (b) Bar graph of FL response of **2** towards different metal ions (λ_max_ = 546 nm). (c) 3D plane of luminescent intensities for **2** in aqueous solution with various metal ions.

(a)

(b)

(c)

**Figure S14.** (a) 2D plane of luminescent intensities of **3** in aqueous solution with various metal ions. (b) Bar graph of FL response of **3** towards different metal ions (λ_max_ = 616 nm). (c) 3D plane of luminescent intensities of **3** in aqueous solution with various metal ions.

(a)

(b)

(c)

**Figure S15.** (a) 2D plane of luminescent intensities of **4** in aqueous solution with various metal ions. (b) Bar graph of FL response of **4** towards different metal ions (λ_max_ = 546 nm). (c) 3D plane of luminescent intensities of **4** in aqueous solution with various metal ions.

(a)

(b)

(c)

**Figure S16.** (a) 2D plane of luminescent intensities of **6** in aqueous solution with various metal ions. (b) Bar graph of FL response of **6** towards different metal ions (λ_max_ = 546 nm). (c) 3D plane of luminescent intensities of **6** in aqueous solution with various metal ions.


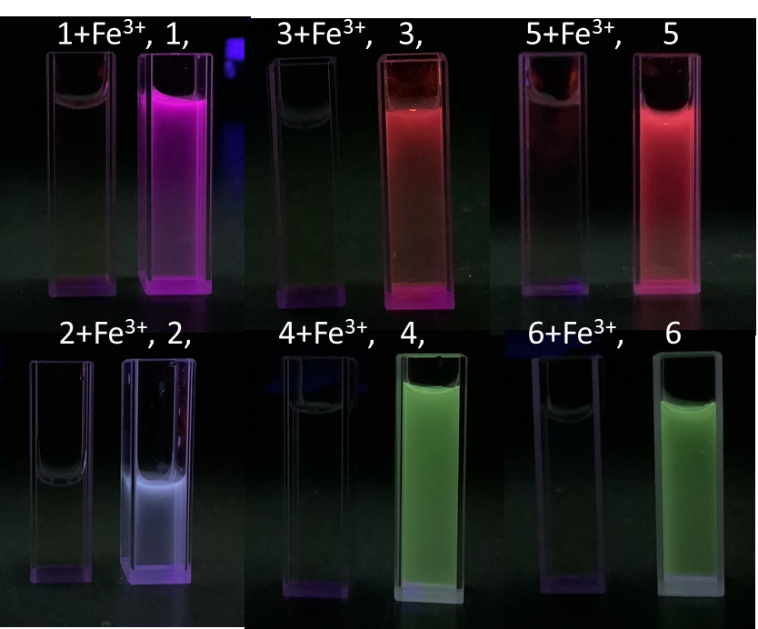


**Figure S17**. The comparative photographs of complexes **1-6** and **1-6** with Fe^3+^ under 254 nm UV light.

**Figure S18.** Fluorescence intensity of **2** dispersed in aqueous solutions of mixed cations without or with Fe^3+^.

**Figure S19.** Fluorescence intensity of **3** dispersed in aqueous solutions of mixed cations without or with Fe^3+^.

**Figure S20.** Fluorescence intensity of **4** dispersed in aqueous solutions of mixed cations without or with Fe^3+^.

**Figure S21.** Fluorescence intensity of **6** dispersed in aqueous solutions of mixed cations without or with Fe^3+^.

(a)

(b)

(c)

**Figure S22.** (a) Luminescence spectra of **2** dispersed in aqueous solutions of FeCl_3_ (10^−2^-10^−8^ M, 10 mL H_2_O, 2 mg **2**). (b) Luminescence spectra of **2** dispersed in aqueous solutions of FeCl_3_ (10^−3^-10^−4^ M, 10 mL H_2_O, 2 mg **2**). (c) Linear dependence between the quenching efficiency and the concentration of Fe^3+^ in the range of 1−10 mM.

(a)

(b)

(c)

**Figure S23.** (a) Luminescence spectra of **3** dispersed in aqueous solutions of FeCl_3_ (10^−2^-10^−8^ M, 10 mL H_2_O, 2 mg **3**). (b) Luminescence spectra of **3** dispersed in aqueous solutions of FeCl_3_ (10^−3^-10^−4^ M, 10 mL H_2_O, 2 mg **3**). (c) Linear dependence between the quenching efficiency and the concentration of Fe^3+^ in the range of 1−10 mM.

(a)

(b)

(c)

**Figure S24.** (a) Luminescence spectra of **4** dispersed in aqueous solutions of FeCl_3_ (10^−2^-10^−8^ M, 10 mL H_2_O, 2 mg **4**). (b) Luminescence spectra of **4** dispersed in aqueous solutions of FeCl_3_ (10^−3^-10^−4^ M, 10 mL H_2_O, 2 mg **4**). (c) Linear dependence between the quenching efficiency and the concentration of Fe^3+^ in the range of 1−10 mM.

(a)

(b)

(c)

**Figure S25.** (a) Luminescence spectra of **6** dispersed in aqueous solutions of FeCl_3_ (10^−2^-10^−8^ M, 10 mL H_2_O, 2 mg **6**). (b) Luminescence spectra of **6** dispersed in aqueous solutions of FeCl_3_ (10^−3^-10^−4^ M, 10 mL H_2_O, 2 mg **6**). (c) Linear dependence between the quenching efficiency and the concentration of Fe^3+^ in the range of 1−10 mM.


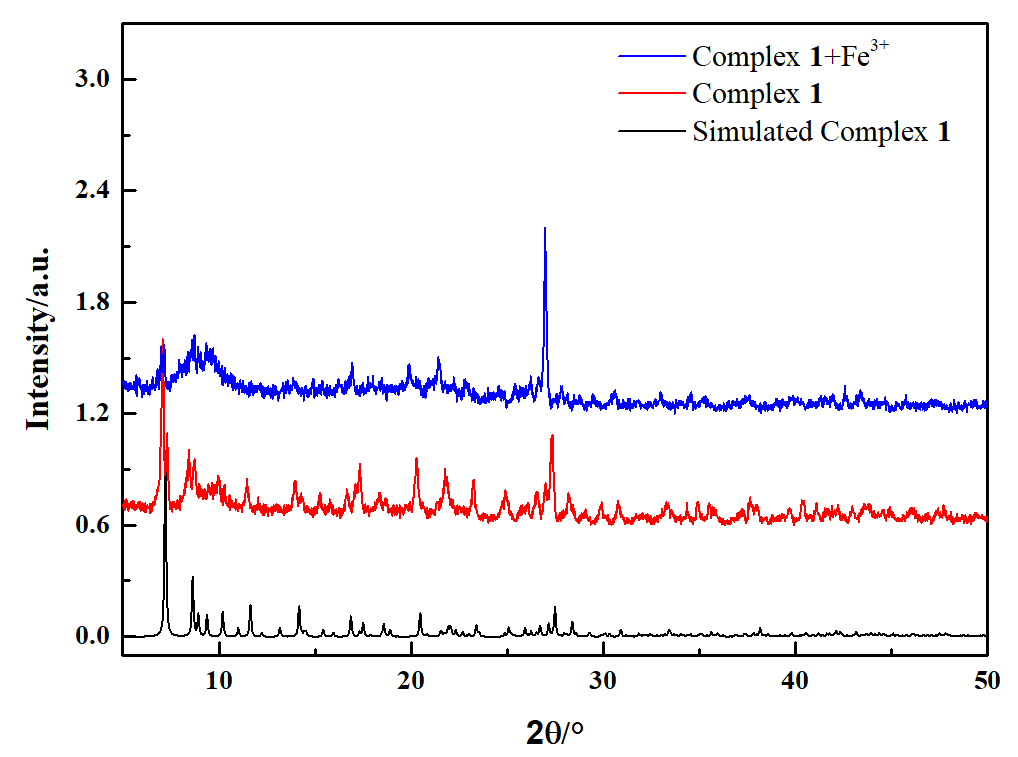

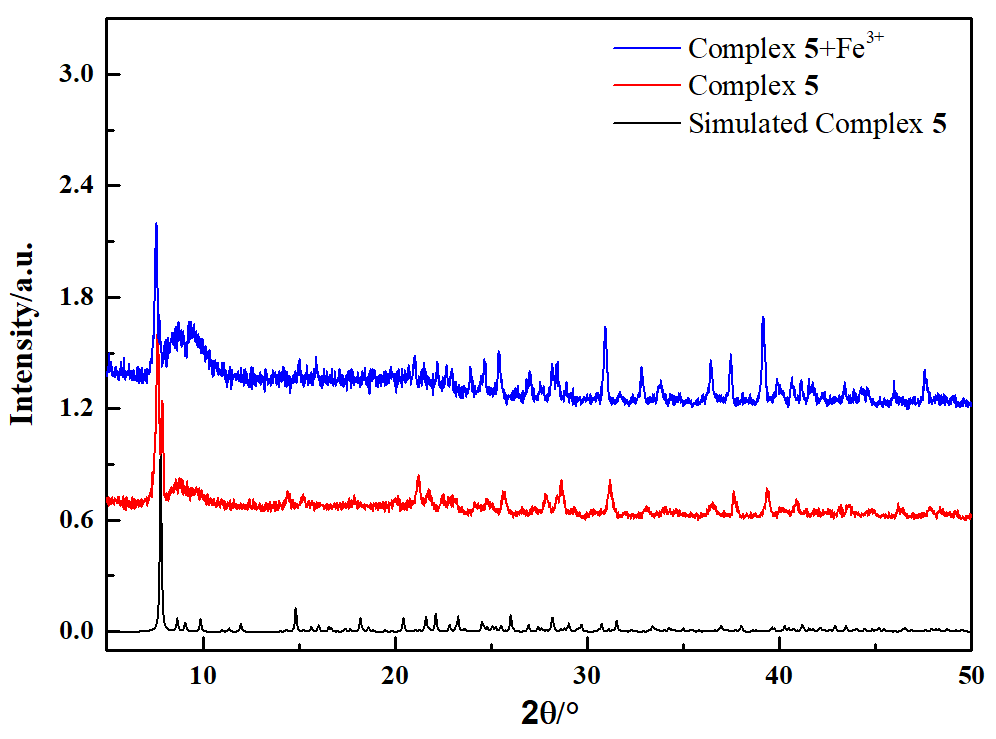


**Figure S26**. The XRD patters of complexes **1** and **5**, before and after suspension in Fe^3+^ ion solution


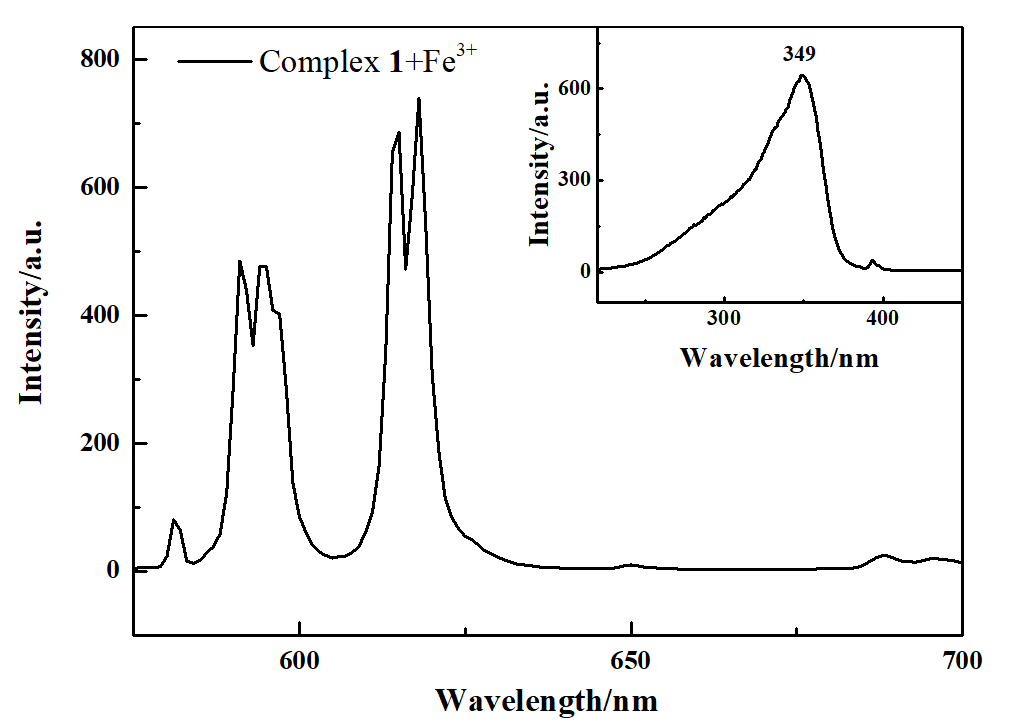

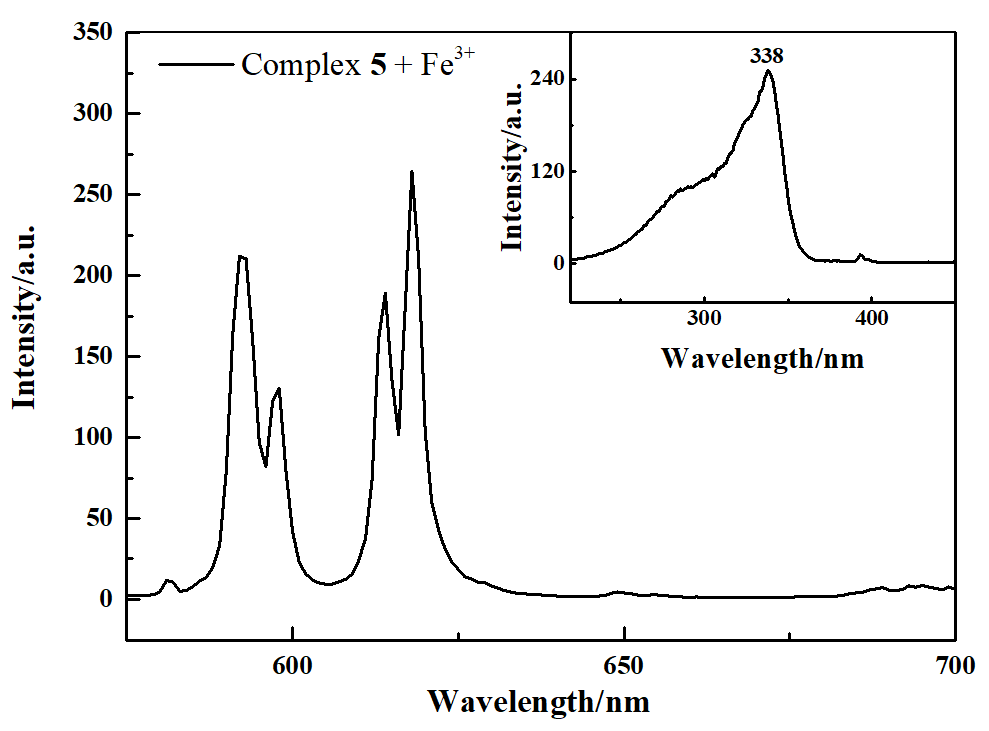


**Figure S27**. Solid state PL spectra of complexes **1** and **5**, tested after suspension of Fe^3+^ solution and repeated rinsing.


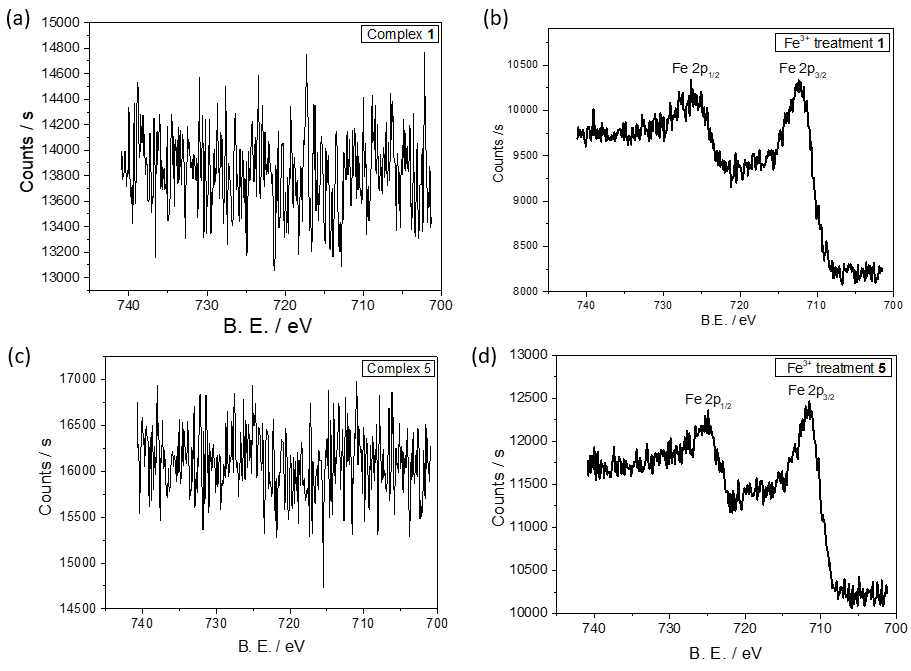


**Figure S28.** The XPS spectra of Fe 2p from the fractured surface of **1**, Fe^3+^ treated with **1**, **5**, Fe^3+^ treated with **5**.


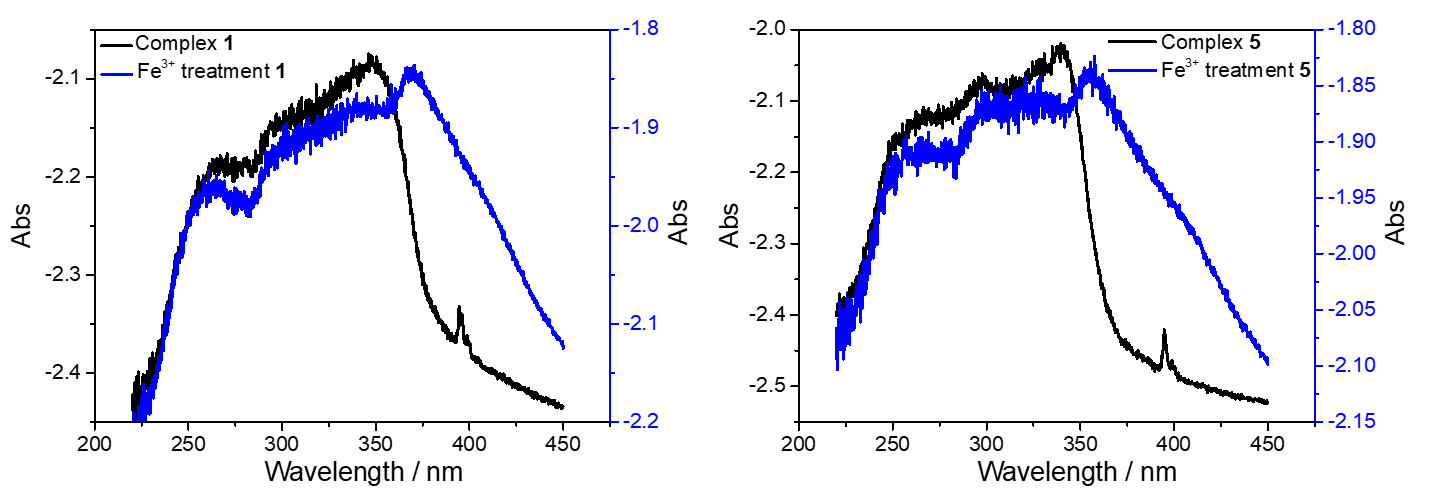


**Figure S29**. The Solid-state UV−vis spectra of 1 and 5 before (black line) and after the treatment with Fe^3+^ (blue line) ions.


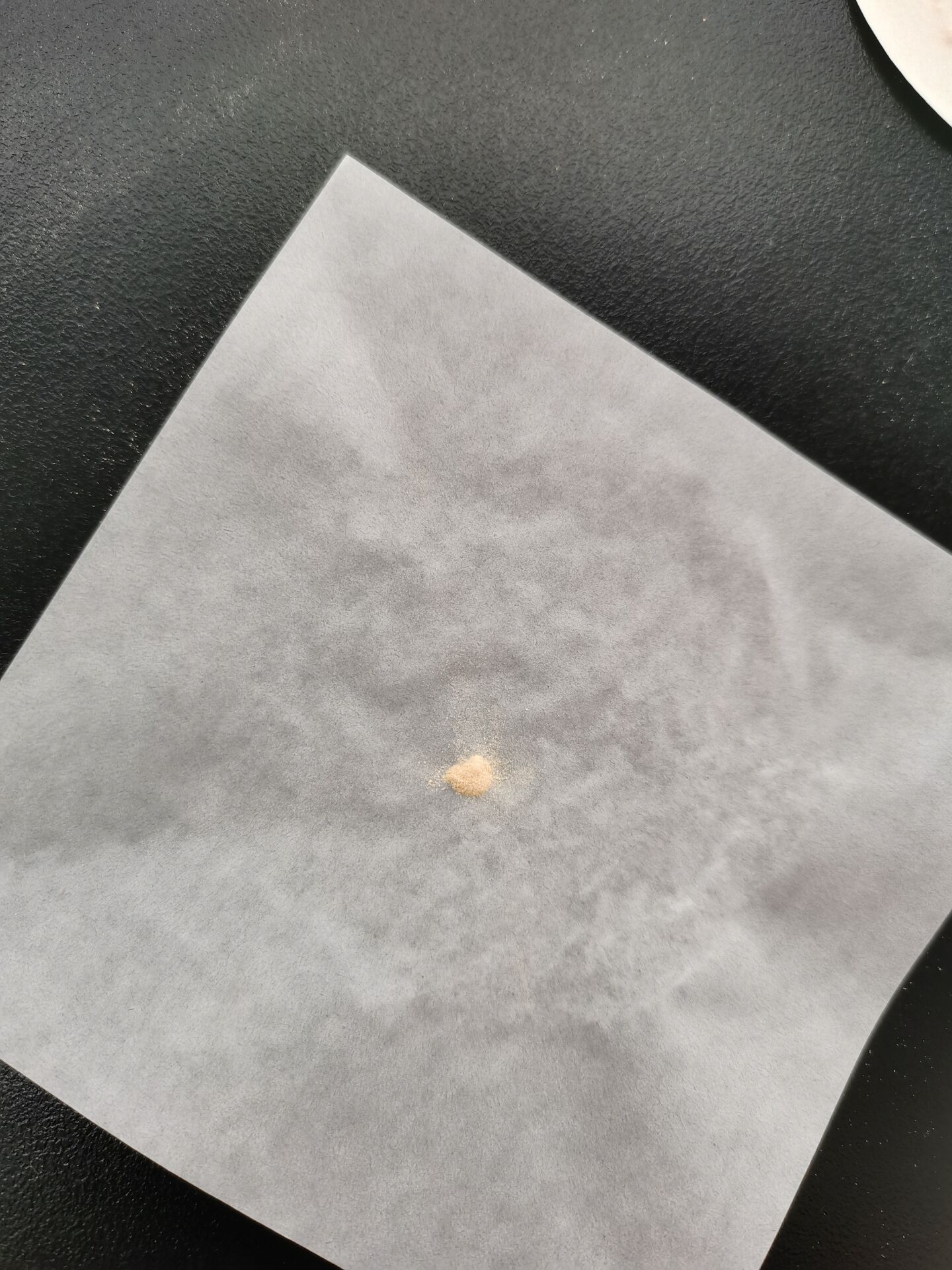

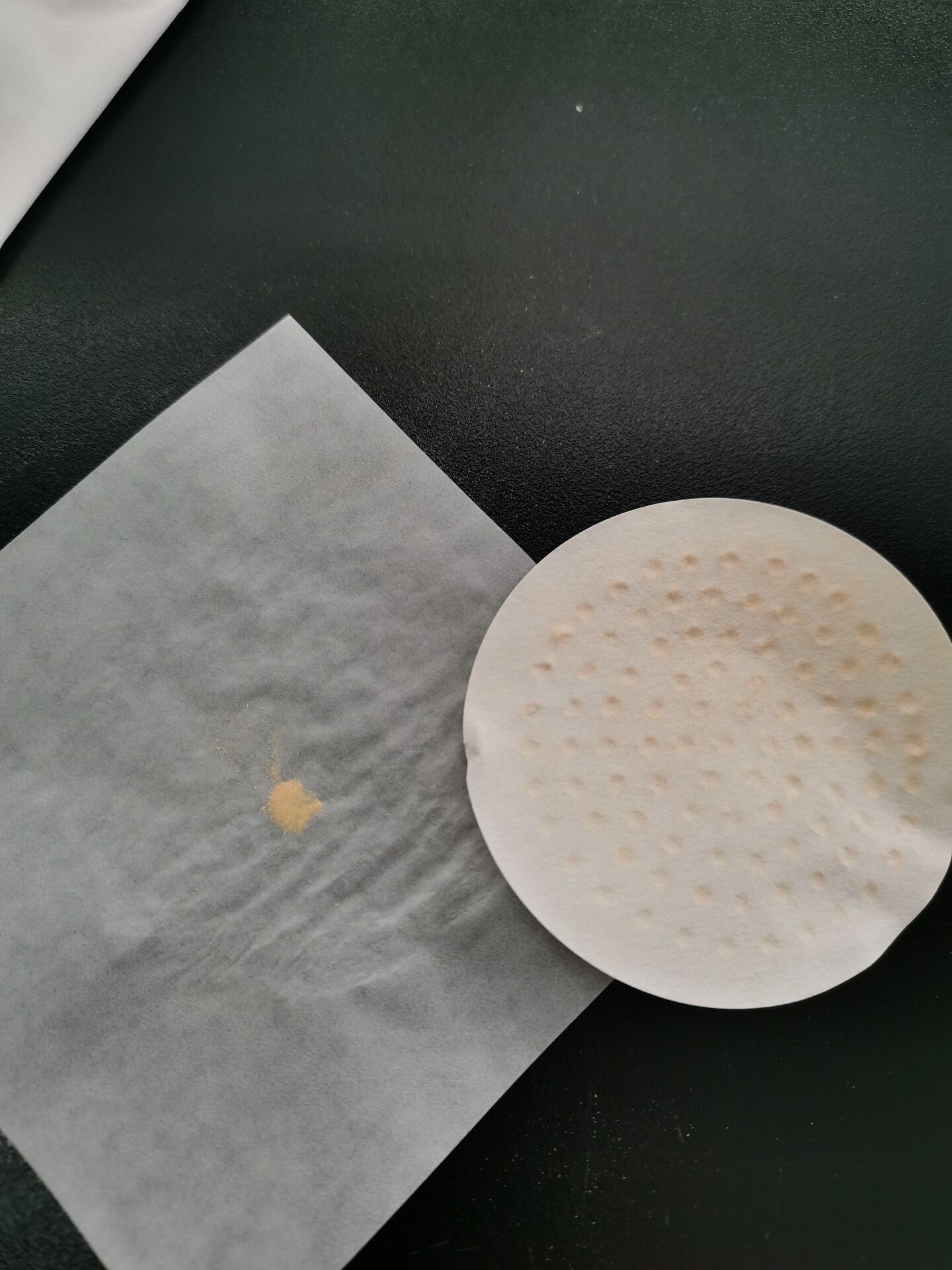


**Figure S30.** The photograph of Fe^3+^ treatment **1** (left) and **5** (right)


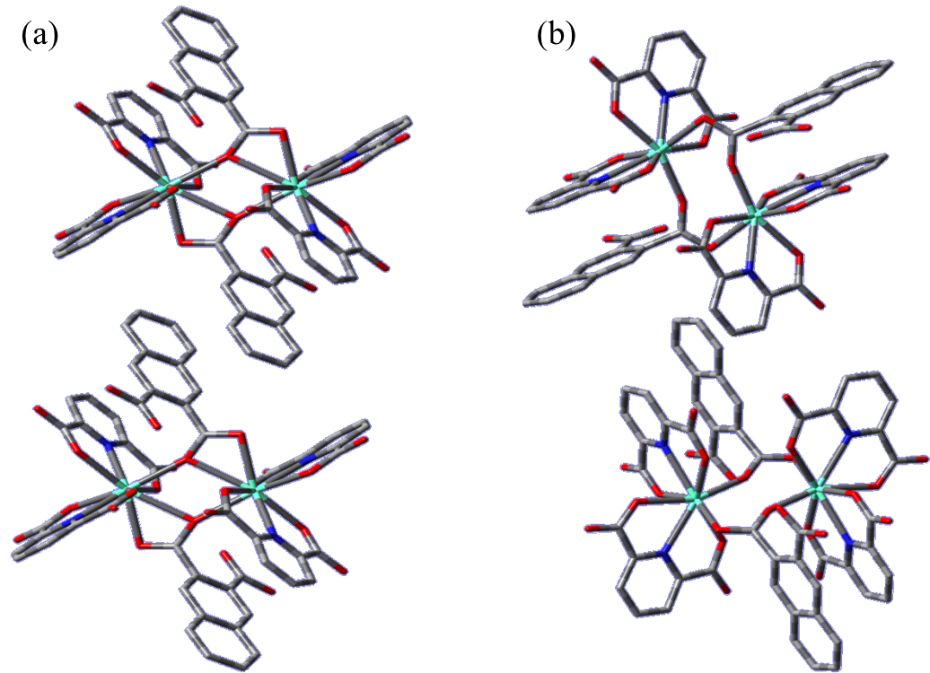


**Figure S31**. The packing configurations of dimers of complexes **1** (a) and **5**(b). The hydrogen atoms are not shown for clarity.
